# How Wearable Sensors Can Support Parkinson's Disease Diagnosis and Treatment: A Systematic Review

**DOI:** 10.3389/fnins.2017.00555

**Published:** 2017-10-06

**Authors:** Erika Rovini, Carlo Maremmani, Filippo Cavallo

**Affiliations:** ^1^The BioRobotics Institute, Scuola Superiore Sant'Anna, Pontedera, Italy; ^2^U.O. Neurologia, Ospedale delle Apuane (AUSL Toscana Nord Ovest), Massa, Italy

**Keywords:** Parkinson's disease, wearable sensors, motion analysis, early diagnosis, tremor, motor fluctuations, monitoring, telemedicine

## Abstract

**Background:** Parkinson's disease (PD) is a common and disabling pathology that is characterized by both motor and non-motor symptoms and affects millions of people worldwide. The disease significantly affects quality of life of those affected. Many works in literature discuss the effects of the disease. The most promising trends involve sensor devices, which are low cost, low power, unobtrusive, and accurate in the measurements, for monitoring and managing the pathology. Objectives: This review focuses on wearable devices for PD applications and identifies five main fields: early diagnosis, tremor, body motion analysis, motor fluctuations (ON–OFF phases), and home and long-term monitoring. The concept is to obtain an overview of the pathology at each stage of development, from the beginning of the disease to consider early symptoms, during disease progression with analysis of the most common disorders, and including management of the most complicated situations (i.e., motor fluctuations and long-term remote monitoring). Data sources: The research was conducted within three databases: IEEE Xplore®, Science Direct®, and PubMed Central®, between January 2006 and December 2016. Study eligibility criteria: Since 1,429 articles were found, accurate definition of the exclusion criteria and selection strategy allowed identification of the most relevant papers. Results: Finally, 136 papers were fully evaluated and included in this review, allowing a wide overview of wearable devices for the management of Parkinson's disease.

## Introduction

Parkinson's disease (PD) is a complex neurodegenerative disorder that has a usually asymmetric onset, characterized by typical motor symptoms as bradykinesia, hypo-/akinesia, muscular rigidity, and resting tremor (Fahn, 2008). Although the pathology is generally diagnosed on the basis of these motor symptoms, many non-motor manifestations (NMMs) are commonly evident and they may sometimes be more disabling of motor disturbances, such as olfactory disturbances, autonomic dysfunction, sleep fragmentation, depression, and dementia (Wolters, 2008). Some NMMs (e.g., sleep disorders, bladder disturbances, gastrointestinal symptoms, olfactory symptoms) may occur throughout the entire course of the disease, even if cognitive symptoms such as hallucinations and dementia tend to occur late in the PD. The disease is difficult to detect and treat promptly, as it shows a wide variability in the clinical expression (Fahn, 2008) as well as in the somatic symptom progression (Dickson and Grünewald, 2004; Caslake et al., 2013; de Lau et al., 2014; Szewczyk-Krolikowski et al., 2014). Over the past three decades, the knowledge of PD has increased significantly, with particular interest on the pre-motor phase and novel therapeutic and diagnostic approaches (Korczyn and Gurevich, 2010). Currently, experts recognize the need to redefine the research criteria for the diagnosis of this complex disease by considering clinical features, pathological findings, and genetics or molecular mechanisms (Mirelman et al., 2011; Berg et al., 2013). Recent studies demonstrate that several NNMs (e.g., rapid eye movement sleep behavior disorders, hyposmia, constipation, depression) are correlated to the neuropathological changes in the brain and they can anticipate the motor manifestations of the disease by 5–7 years. Furthermore, the study of the pre-motor phase could lead the research for predictive biomarkers and risk or protective factors for PD (Tolosa and Pont-Sunyer, 2011; Palma and Kaufmann, 2014). Today, PD diagnosis is based on the assessment of motor (and non-motor) symptoms, typically during neurological visual examinations, but the diagnostic methods and disease progression monitoring approaches remain suboptimal for PD management (Kassubek, 2014). This is particularly true when co-factors such as greater age, poor cognition, and worse mobility are manifested (Hu et al., 2011). During the test for PD diagnosis, in fact, the neurologist watches the patient perform specific tasks and assigns scores for each of them as required and defined in the Unified Parkinson's Disease Rating Scale (UPDRS) (Fahn and Elton, 1987) or its updated version, the Movement Disorder Society-sponsored revision of the UPDRS (MDS-UPDRS) (Goetz et al., 2008). The Hoehn and Yahr scale (HY) (Hoehn and Yahr, 1967) instead includes stages 1–5, and it is used to assign an overall score to the patient on the basis of the pathological progress. All these clinical scales are subjective; this fact leads to high inter-rater variability among different neurologists or different medical centers, as well as high intra-rater variability over time. The correct diagnosis of PD is of vital importance for adequate prognosis and treatment, although a study reveals that ~25% of diagnoses are incorrect, particularly when essential tremor, vascular Parkinsonism, and atypical Parkinsonian syndromes are manifested (Tolosa et al., 2006). An exhaustive study of the pathology, including a more accurate knowledge of its clinical appearance and other tests such as olfactory exam and magnetic resonance imaging (MRI), could guide the correct diagnosis (Tolosa et al., 2006). The treatment for PD is still a matter of debate, especially in the early phases. Common sense says that the therapy must be personalized and adapted to the individual needs of PD patients to provide the best medical care and treat the predominant symptoms (Ossig and Reichmann, 2015). Early and accurate diagnosis of PD may improve the long-term quality of life (QoL) for PwPD, while misdiagnosing a patient causes delay in receiving the appropriate treatment plan.

In this context, the use of smart technologies for PD applications has increased in recent years. In particular, wearable sensors are fundamental in helping clinicians perform early diagnosis, differential diagnosis, and objective quantification of symptoms over time. A growing number of papers concerning this topic during the last decade also demonstrate the increasing development and use of such wearable technologies. For example, the use of inertial sensors such as accelerometers (ACC) and gyroscopes (GYRO), combined with advances in short-range communication technologies (i.e., Bluetooth, Zigbee), is now feasible and meets the needs of people with chronic disorders by featuring low power consumption, unobtrusiveness, light weight, and ease of use (Bonato, 2010). Wearable sensors have demonstrated their potential power for PD diagnosis (Butt et al., 2017) and management (Rovini et al., 2016), as well as for other pathologies (e.g., post-stroke, neck injuries) (Rodgers et al., 2015) or to monitor pharmacological trials (Henderson et al., 2016). In terms of pharmacological treatment, levodopa (Ldopa) is currently the most used and effective medication for PD, even if several side effects result from it, especially motor fluctuations and dyskinesias (Chou, 2008). When Ldopa-related side effects are difficult to control, surgical therapies such as neuromodulation using deep brain stimulation (DBS) (Rissanen et al., 2015) can be applied, while at the same time, other potential solutions (e.g., biological therapies) are emerging (Strauss et al., 2014). To redefine new metrics for early diagnosis, differential diagnosis, and quantification of symptoms, the development of a system for objective assessment of the pathology to identify motor dysfunctions, which are imperceptible upon expert clinical exam, is required (Scanlon et al., 2013). Finally, it is important to consider also the social aspects that are involved with a disabling pathology such as PD. The burden of care among caregivers of PwPD considerably increases with age and disease progression and is linked to the period and level of assistance required (Razali et al., 2011), whereas non-motor symptoms, especially cognitive decline, play a prevalent role in caregivers' grief (Carter et al., 2012). To reduce the burden of caretakers, a recent study (Megalingam et al., 2014) proposes a wearable health monitoring system that can measure heart rate, temperature, electrocardiogram (ECG), tilt, and fall of the homebound patients and can send a notification via smartphone to the caregivers if a critical situation is occurring. Such a system would enable remote assistance.

The aim of this review is to provide readers with broad scientific and technological information about the use and challenges of wearable sensor technologies for PD applications. This paper details the investigation of the typology of wearable sensors, fields of application, processing approaches, and experimental methodologies. Such a complete overview of PD wearable technology makes this paper highly suitable for scientists with both clinical and technical background. In particular, this paper provides a review of the typologies of wearable sensors that were investigated and adopted for PD applications in the last decade, and it describes implemented experimental protocols, the subjects of the studies, extracted features, and performance of classifiers. Such wearable technologies are organized with respect to five critical fields of application that cover the entire pathology progression: (1) early diagnosis, (2) tremor, (3) body motion analysis, (4) motor fluctuations and ON–OFF phases, and (5) home and long-term monitoring. For each topic, the existing systems, found using the methodology described in the next paragraph, were investigated. The results are presented here, and recommendations for further development and discussion of future trends are provided as well.

## Methods

### Search strategy

An electronic database search was performed from September 2016 to December 2016 using IEEE Xplore®, Science Direct®, and PubMed Central® to identify articles concerning the use of wearable sensors for PD applications. Specifically, the terms and key words used for the literature research were (“*Parkinson*”) AND (“*wearable*” OR “*inertial*” OR “*accelerometer*” OR “*acceleration*” OR “*gyroscope*” OR “*EMG*” OR “*EEG*” OR “*ECG*” OR “*GSR*” OR “*clothes*”) located within title and/or abstract. Only original, full-text articles published in English, between January 2006 and December 2016, which discussed the use of wearable sensors for PD applications, were included in the review. Obtained in the research were 485 references from IEEE Xplore®, 653 references from Science Direct®, and 291 references from PubMed Central®. Five major applications were identified: early diagnosis, tremor detection, analysis of the motor performances, analysis of motor fluctuations (on/off phases), and home and long-term monitoring. Papers were screened from three independent reviewers (i.e., the authors) and disagreements were solved through meetings and discussions. Finally, the selected papers were classified on the basis of the application area. Data abstracted from the papers and reported in Tables 1–**9** considered: the used technological solutions and typology of sensors, their placement over the body and the sampling frequency; the experimental protocol adopted; the subjects involved, according to their pathology and their health status; the performed analysis, including the extracted features, the applied statistical methods, the implemented classifiers and the main findings for each work. Particular attention was focused on the classifiers performance because they can synthetically represent the robustness of the technology proposed for a specific PD application.

**Table 1:** Papers about early diagnosis.

**References**	**Tech**.	**Sensor place**	**Rec. freq**	**Experimental design**	**Subjects**	**Feature extracted**	**Analysis/classifiers**	**Classifier performance or findings**
Chen et al., 2014	ACC, GYRO	Trunk	Not reported	Upright standing position, arms crossed on the chest, looking ahead (each 30 s): (i) eyes open (EO), (ii) feet together eyes closed (EC), (iii) feet together EO dual task (EODT), (iv) EC dual task (ECDT), (v) tandem standing EO (TEO)	24 PwPD -SBD, 23 HC	RMS ACC and Jerkiness of sway (Jerk) in mediolateral (ML) and anteroposterior (AP) directions	ANOVA, *t*-Test, Pearson Chi Square test	Differences in Jerk between PD/HC for EODT (*p* = 0.030), ECDT (*p* = 0.015) and TEO (*p* = 0.023)
Mancini et al., 2011	ACC, GYRO	Trunk	100 Hz	Posture: (i) EO gaze straight ahead at an art poster 6 m ahead; (ii) EC, upright standing position; (iii) EC, cognitive task (ECT)	13 PwPD *de novo*, 12 HC	Jerk, RMS of displacements, mean velocity, frequency (freq) below which is 95% of the power of the COP displacement power spectra (F95%), freq dispersion (FD)	Linear mixed model; ROC	AUC: 0.90 for F95%, 0.87 for FD, 0.82 for RMS, 0.93 for Jerk (EO) for untreated PD/HC classification. No significant correlation with UPDRS III
Sant'Anna et al., 2011	GYRO	Wrists, shanks	200 Hz	Walking 30 m hallway, preferred speed (2 min)	11 early/mild PwPD, 15 HC	Symmetry indexes	*t*-Test, ROC; ICC	0.872 AUC for PD/HC classification; ICC = 0.949
Perumal and Sankar, 2016	Force sensors	Below toe, toe	100 Hz	Gait and Tremor (60 s)	Gait: 93 PwPD (HY 2/3), 73 HC; Tremor: 16 PwPD	*Gait*: Step length, stride time, stance time, swing time, heel, below toe and toe forces and normalized heel, below toe and toe forces. *Tremor*: amplitude, power distribution, FD, median freq	ANOVA; LDA 5-fold cross validation; ROC	*Gait*: mean acc.: 91.58%, ROC 0.72 sens., 0.81 spec. and AUC 96% for PD/HC classification. *Tremor*: features able to distinguish PD tremor from atypical PD tremor

### Selection criteria

**Figure 1:**
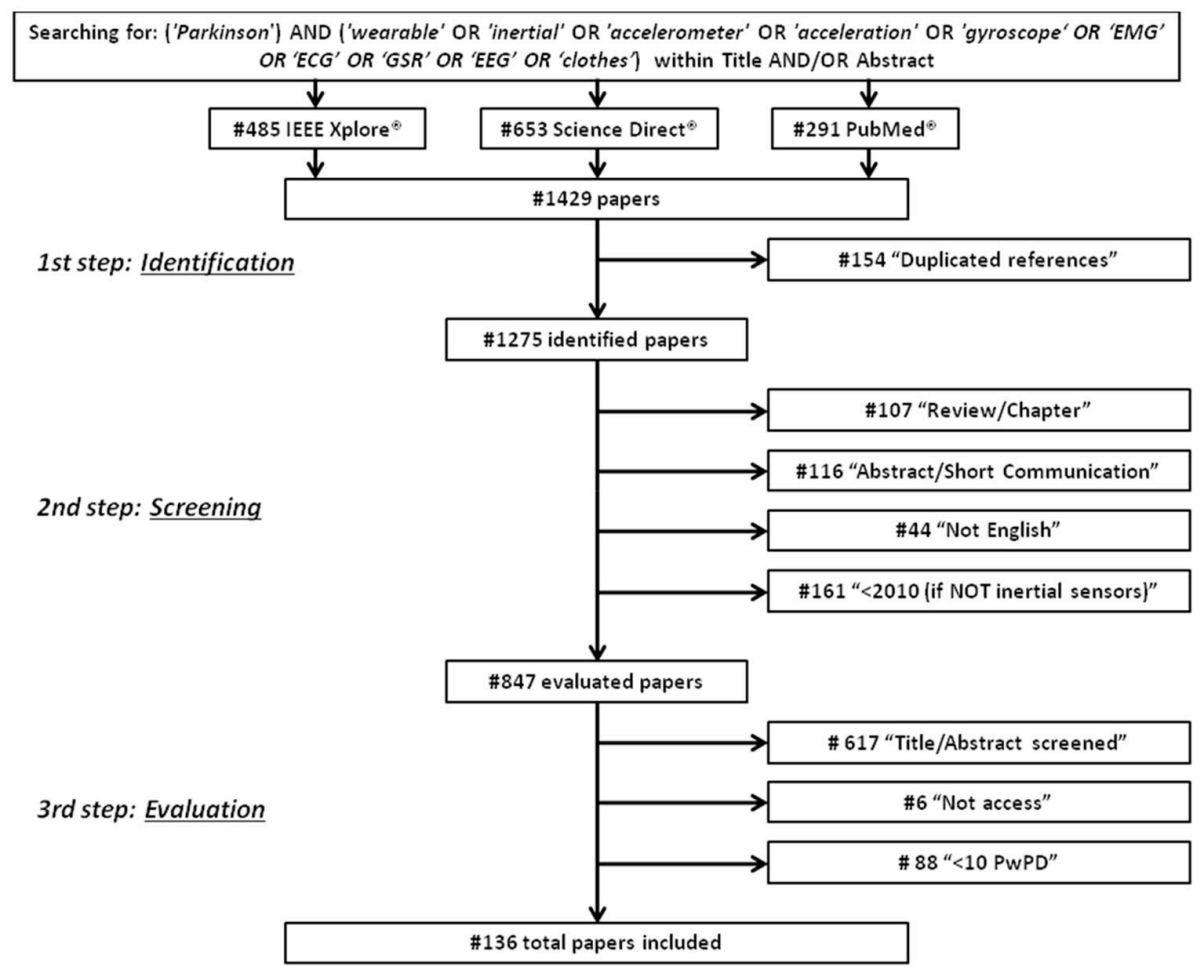
Research methodology for review process.

First, duplicated references were excluded. Then, during the screening procedure, items were excluded if they (i) were an abstract, a short communication, a review article, or a chapter from a book; (ii) were not written in the English language; (iii) were from years prior to 2010 only for sensors other than inertial (i.e., EMG, EEG, ECG, GSR) because they did not concern wearable devices. Eight hundred and forty-seven references were fully assessed during the evaluation procedure, and papers were excluded if (1) they did not use any type of wearable sensors; (2) they did not appear appropriate for this review after the reading of title and abstract; (3) they did not involve patients with Parkinson's Disease; (4) they were not full access; and (5) they involved a number of PD patients <10, due to the low level of reliability and statistical validity that can be obtained from their results. In addition, if multiple papers written by the same author had similar content, papers published in journals were selected instead of papers presented at conferences. Furthermore, if multiple papers written by the same author with similar content were presented at conferences, the most recent paper was selected. Finally, 136 papers were fully evaluated and included in this review (Figure 1).

## Results

**Figure 2:**
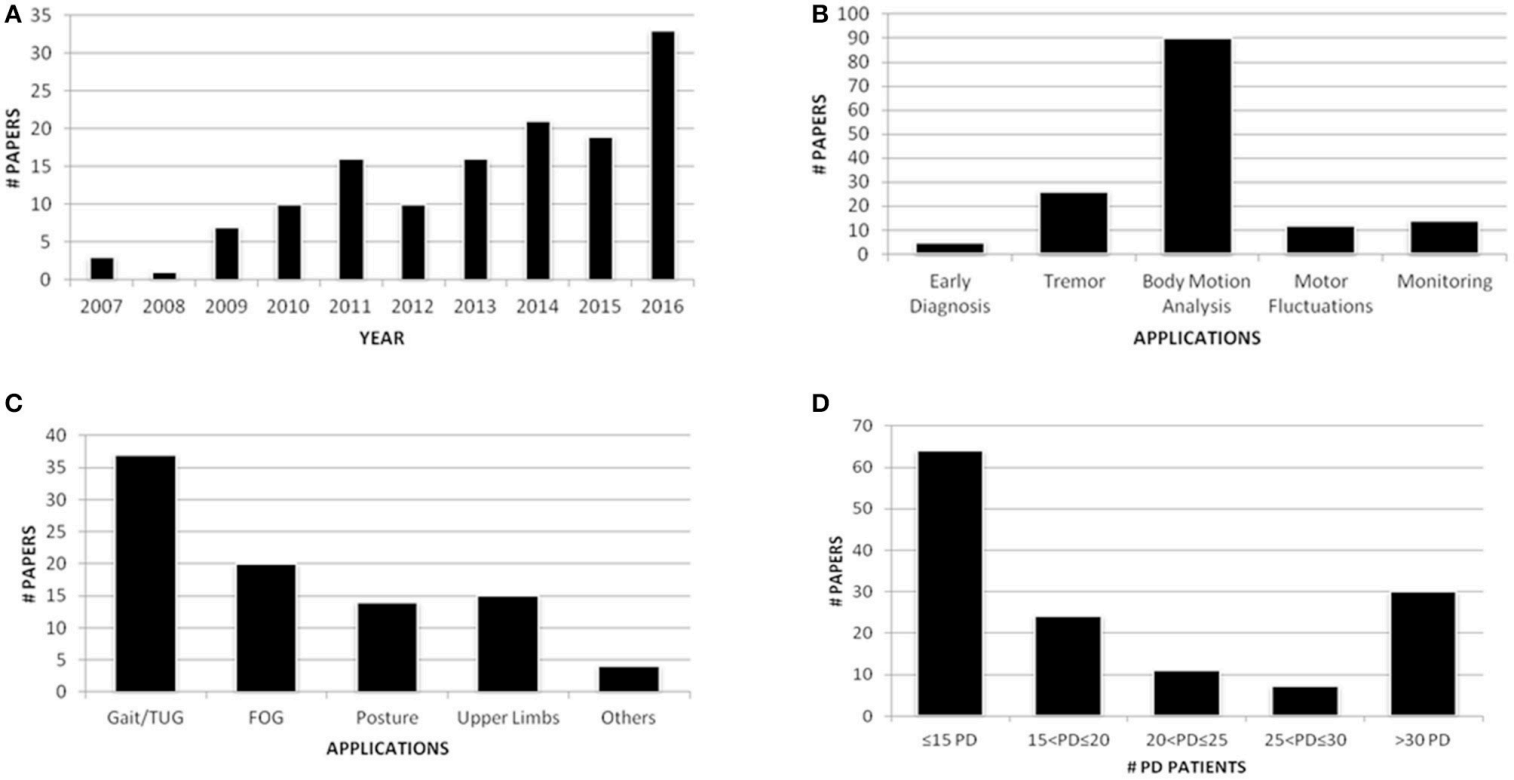
Publication trend per year **(A)**; paper distribution per application **(B)**; papers within body motion analysis application **(C)**; PD patients involved in the studies **(D)**.

Of the 136 fully evaluated papers, 33 (24.3%) were published in 2016, and 73 (53.7%) were published during the last 3 years. This result confirmed the increasing interest for wearable sensors in PD applications (Figure 2A). Eleven papers were appropriate for further applications, which resulted in a total of 147 papers. Among the applications covered by this review, the majority of the papers (61.2%) focused on body motion analysis (Figure 2B) and in particular on gait analysis, which resulted in the most investigated task (37 papers, 25.2%) (Figure 2C). Finally, regarding the number of PD patients involved in the studies, even if works that recruited less than 10 PwPD were excluded from this review, the majority of the research efforts (47.1%) included fewer than 15 PwPD (Figure 2D). Thus, clinical validation of the proposed solutions is still a matter of debate. Because of the significant number of papers included in the review process, only papers published in journals (58.8%) will be shown in detail in the tables presented here, whereas the full dataset of articles, including 56 papers from conferences, will be uploaded as Supplementary Data online.

### Application 1: Early diagnosis

Only 5 papers that deal with early diagnosis were obtained by following the research criteria of this review (Table 1). Posture detection systems were investigated because symptoms of postural instability are frequent in the early stage of PD and can lead to complications from festination in the next phases of the pathology. Postural sway performance seems to be a biological marker for prodromal PD (Chen et al., 2014), as it results in an abnormal quite stance in subjects with untreated PD (Mancini et al., 2011).

In contrast, Brodie et al. (2014) proposed to analyse new features extracted from gait, which could represent a biomarker for PD. Jerk, harmonic stability, and oscillation range measured by accelerometers on the pelvis and head were significant measures to distinguish early PwPD when compared to similar measurements for age-matched healthy control (HC) subjects. Sant'Anna et al. (2011) also analyzed gait, focusing on both leg movements and arm swing. They demonstrated that asymmetry between left and right sides in PwPD is higher than in HC, particularly for upper limbs. Different indexes for asymmetry assessment proposed in literature were compared, since this characteristic results in one of the first motor symptoms of the disease. Perumal and Sankar (2016) also considered selected features extracted by gait analysis as possible biomarkers for early diagnosis of PD, because the features enabled good discrimination between PwPD and HC. Perumal and Sankar also analyzed tremor in the frequency domain to differentiate between Parkinsonian tremor and atypical Parkinsonism. They found that different typos of tremor occurred in different frequency bands; particularly, resting tremor occurred during the early stages of the disease, appearing at the onset in approximately 70% of patients.

#### Recommendations and trends

The research for technological solutions able to address the early identification of PD is lacking, as demonstrated by the small number of papers found according to the inclusion criteria of this review. The works specifically focused on posture, gait, and asymmetry analyses. The idea is to recognize pathologically abnormal postures as soon as possible, from the prodromal phase of the pathology (Chen et al., 2014), to allow intervention at the earliest stage of PD. To obtain accurate results, the studies must be validated on a large dataset, involving a wide number of subjects with PD at only a mild idiopathic clinical stage of the disease (Brodie et al., 2014). The study of Perumal and Sankar (2016), for example, aimed to accomplish early diagnosis, but it involved PwPD with HY stage 2–3, which is an inappropriate dataset of patients. To investigate the early stage of the disease, only subjects with minimal motor abnormalities must be included in the studies (Sant'Anna et al., 2011), even if recruitment of such patients is difficult because they often do not go to the doctor until symptoms are already widely manifested. In this sense, large prevention and screening programs to identify patients at risk to develop the disease should be recommended and investigated, promoting the early diagnosis of pathology with positive and effective consequences on the therapeutic treatments.

### Application 2: Tremor

Tremor is the most common symptom of PD, resulting in 26 papers in this review; it appears in 70% of patients and typically involves one side only, at the beginning. Resting tremor (RT) is the prevalent type, and it appears generally when limbs are not intentionally moved, when patient is sitting (typically pill-rolling tremor of the hands) or walks with arms dangling. Other typos of tremor are postural tremor (PT) that occurs when the body part is contracted against gravity, kinetic or action tremor (KT) that is task-specific, limited to duration of performing a particular task (e.g., writing), essential tremor (ET) which can overlap the frequency band of RT but is associated with a movement disorder different from PD, and physiological tremor which is present in healthy subjects. Tremor is a complex cerebello-thalamo-cortical phenomenon, but the specific role of the cerebellum in suppressing or generating tremor remains unclear. As the assessment of tremor is currently based on the visual examination of a neurologist, technological solutions able to quantify the gravity of the disease, and efficacy of the therapy appear to provide an optimal solution that offers low invasiveness and high reliability (Scanlon et al., 2013). Correct diagnosis of different tremors is important because the treatment depends on the specific etiology of each tremor type. However, currently reported misdiagnosis between RT and ET may occur in 20–30% of the cases (Ghassemi et al., 2016; Surangsrirat et al., 2016). In literature, several works have analyzed the use of an inertial measurement unit (IMU) and other sensors, such as electromyography (EMG), which can be complementary in detecting tremor. These sensors can be attached to different parts of the body (e.g., ankles, shanks, lower back) to measure the tremor in PwPD (Table 2). Further works proposed alternative solutions, such as smart clothes (Niazmand et al., 2011) or wearable glove systems, but the datasets for the experiments with these gloves were limited (≤5 PwPD) so were not analyzed in this review. In contrast, Bazgir et al. (2015), Kostikis et al. (2015), and Daneault et al. (2012) proposed smartphone-based systems that were mounted in a custom-made glove-case from which the acceleration signal was recorded. Finally, Braybrook et al. (2016) proposed the Parkinson's Kinetigraph System, a wrist-worn device able to collect data continuously over an extended time period and detect tremor events. Data for tremor analysis were collected while the subjects performed standard diagnostic exercises according to UPDRS for assessment of RT, PT, KT; or conducted a reaching task (Alhamid et al., 2010) or an action task (Kwon et al., 2016). In these exercises, it is crucial to recognize different typos of tremors (e.g., essential, resting, postural, re-emergent) accurately (Thanawattano et al., 2015; Surangsrirat et al., 2016), analyse the various frequency bands properly (Hossen et al., 2010, 2013; Niazmand et al., 2011; Daneault et al., 2012; Hossen, 2012; Rigas et al., 2012, 2016; Pierleoni et al., 2014; Bazgir et al., 2015; Ghassemi et al., 2016; Kwon et al., 2016; Mailankody et al., 2016; Zhou et al., 2016), and distinguish tremor correctly from other movements and disorders (e.g., dyskinesias, bradykinesia) (Salarian et al., 2007b; Rigas et al., 2012, 2016; Pierleoni et al., 2014), as well as recognize tremor severity accurately (Salarian et al., 2007b; Daneault et al., 2012; Rigas et al., 2012; Pierleoni et al., 2014; Bazgir et al., 2015). For these purposes, a frequency analysis was the most appropriate approach (Salarian et al., 2007b; Daneault et al., 2012; Hossen, 2012; Cavallo et al., 2013; Scanlon et al., 2013; Pierleoni et al., 2014; Bazgir et al., 2015; Braybrook et al., 2016; Zhou et al., 2016), and subjects with tremors in their hands were expected to have higher power in the high-frequency components (Alhamid et al., 2010). Although most of the works used signals from accelerometers to calculate features for tremor assessment, Surangsrirat et al. (2016) and Thanawattano et al. (2015) proposed the use of angular velocities to calculate the ratio of temporal fluctuations of tremor signal during resting tasks and kinetic tasks. This method can differentiate between PD tremor and ET since PwPD have a potential for higher tremor fluctuations with PD tremors. Additionally, Salarian et al. (2007b) used only gyroscope signals to calculate mobility and activity of the hand during selected time windows.

**Table 2:** Papers about tremor analysis.

**References**	**Tech**.	**Sensors place**	**Rec freq**	**Experimental design**	**Subjects**	**Feature extracted**	**Analysis/classifiers**	**Classifier performance or findings**
Scanlon et al., 2013	ACC, Tremor Pen™	ACC: Feet. Pen: hand	Not reported	Resting task; Postural task with distracting task [upper and lower limbs, both dexterity dominant (DD) and non-dominant (nD)] (each 8.2 s)	16 PwPD, 8 HC	RMS of ACC data, F0, F50, intraindividual variability of F50 (IIVF50)	Mann-Whitney U and Wilcoxon signed-rank tests	IIVF50 for RT (*p* = 0.032) and PT (*p* = 0.017) lower in the DD lower limb of PwPD compared to HC. RT F50 lower in the lower limbs than in the upper in DD limbs (*p* = 0.008) and nD limbs (*p* = 0.001). RT IIVF50 different between the upper and lower nD limbs (*p* = 0.039)
Kostikis et al., 2015	Smart phone	Custom made glove case	20 Hz	Resting task; Postural task (each 30 s)	23 PwPD, 2 *de novo* PD, 20 HC	Freq (PSD)	Pearson coefficient; Bag DT	0.7 < *r* < 0.87 for RT, *r* < 0.6 for PT for UPDRS correlation (PwPD); Negative % differences because tremor increases in OFF phase (*de novo* PD); 82% sens., 90% spec., 0.94 AUC for PwPD/HC classification
Daneault et al., 2012	Smart phone	Hand	60 Hz	Resting task, Postural task, Intention task, Kinetic task (bringing the phone to one's ear and back at a relatively slow velocity).	12 PwPD, 3 ET, 1 multiple sclerosis	Tremor amplitude, regularity, power distribution (3–7 Hz), median, peak of power freq, power dispersion, harmonic index	Pearson coefficients	Tremor amplitude correlation to clinical scale: *r* = 0.76 for RT; *r* = 0.85 for PT; *r* = 0.88 for tremor amplitude for Intention tremor; *r* = 0.09 for KT. *r* = 0.7 for power distribution in KT
Braybrook et al., 2016	Parkinson KinetiGraph System	Wrist	50 Hz	Continuous recording of tremor between 09:00 and 18:00 for at least 6 days	Cohort 1: 85 PwPD; Cohort 2: 87 PwPD; 22 *de novo* PD	Proportion Tremor Time (PTT): % time of tremor between 9 and 18	ROC, AUC, sensitivity, selectivity	Tremor identification with PTT > 0.8%: 92.5% sens., 92.9% selectivity (AUC = 0.92) in cohort 1; 90.3% sens., 92.7% selectivity (AUC = 0.96) in cohort 2; 88.7% sens., 89.5% selectivity (AUC = 0.95) in combined group. *de novo* PD: 54% had PTT > 0.8%, 50% had PTT > 1.0%
Kwon et al., 2016	2 channel sEMG	Extensor and flexor carpi radialis	Not reported	(i) Resting in a comfortable position, (ii) Postural state, (iii) Action position: writing, spooning, cup-holding	24 PwPD-TDT (tremor dominant), 28 ET	tremor freq and the contraction pattern of agonist–antagonist muscles	Fisher's exact test	Difference PD/ET for number of RT (*p* = 0.005) and co-contraction pattern in KT (*p* = 0.024). PD-TDT had more non-motor symptoms compared to ET (*p* = 0.002)
Rigas et al., 2012	ACC	Wrists, ankles, chest, waist	62.5 Hz	Resting tasks (rest in bed and chair, standing up with the hands resting); Postural task; Kinetic tasks (finger-to-nose, finger to finger, walking, picking up/holding objects)	18 PwPD (10 tremor, 8 other PD symptoms), 5 HC	Dominant freq, amplitude of dominant freq, power spectrum, spectrum entropy, energy	HMM (leave one patient out)	81% acc. for posture/action detection; 87% acc. for tremor severity classification; >95% spec. for tremor/other symptoms discrimination
Hossen et al., 2010	ACC, EMG	Hand, forearm	800 Hz	Postural task (30 s)	Train set: 19 PwPD, 21 ET; Test set: 20 PwPD, 20 ET	PSD, entropy	Not specified	80% sens., 90% spec., 85% acc. for PD/ET classification
Hossen et al., 2013	ACC piezo electric	Dorsum of the more affected hand	Not reported	Postural tremor from the more affected side (30 s)		Mean and mean deviation of amplitude and period based on Statistical Signal Characterization-method	Spec., Sens., Acc.	PD/ET discrimination: 100% acc. on training data; 85% acc. on testing data
Mailankody et al., 2016	EMG	Flexors and extensors of wrist	Not reported	(i) Hands at rest; (ii) hands outstretched; (iii) hands close to chest; (iv) re-emergence of tremor (ReRT) with hands outstretched (>1 min)	63 PwPD: group 1 (26 RT and ReRT), group 2 (37 RT and PT)	Pattern [alternate (alter) vs. synchronous (sync) contraction] and freq of tremor. Duration of silent period	Not specified (significant *p* < 0.05)	Illness shorter in ReT than the others (*p* = 0.03). ReT similar to RT in freq and pattern of contraction. Group 1: pattern of contraction alter in 92% and sync in 8% of patients for RT, alter in 85% and sync in 15% of patients for ReT. Group 2: pattern of contraction alter in 73% and sync in 27% for RT, alter in 84% and sync in 16% of patients for PT
Salarian et al., 2007b	GYRO	Wrists	200 Hz	1st study: 17 tasks from ADLs (45 min); 2nd study: free activities (5 h)	1st^:^ 10 PwPD STN DBS, 10 HC; 2nd 11 PwPD STN DBS	Spatiotemporal (mobility, activity and range of rotation of hand) and freq spectrum	Temor detection algorithm *ad hoc*; Pearson coefficient	99.5% sens., 94.2% spec. for tremor detection compared to video recordings; *r* > 0.8 for UPDRS correlation
Ruonala et al., 2014a	EMG Biomonitor ME6000, ACC	EMG: biceps brachii (BB) muscle ACC: forearm	1,000 Hz	Standing in upright position and holding elbows at a 90° angle with the palms upwards: (i) without additional load, (ii) with 1 kg load, (iii) with 2 kg load (each >15 s).	35 PwPD, 17 ET, 40 HC	EMG signals: single motor unit action potentials at random intervals. ACC: RMS, sample entropy, peak freq	PCA; Spearman coefficient; LDA, *k*-means clustering; Mann-Whitney	PC1 and PC3 best differentiated ET/PD; 75% discrimination PD/ET (excluding rigid patients, 81%); Increasing the load (1–2 kg) decreases the discrimination; Strong positive correlation between UPDRS III and PwPD PC1 (*p* < 0.001). Good discrimination PD/ET, significant differences in RMS, Sample Entropy, peak freq
Hwang et al., 2009	ACC	Index finger, hand, forearm, arm, spine C7	400 Hz	Postural task with and without load (20 s)	11 PwPD, 11 HC	PCs, PSD	ANOVA and *t*-Test	Group effect with larger PT for PwPD for all segments (*p* < 0.001); Significant main effect of load modulation of segment tremors except the C7 ACC; load effect on the variance of PC1 (*p* < 0.05); population effect for variance explained by PC2 (*p* < 0.05)

The principal aim of these works was to find a correlation between the features measured and the clinical scores assigned by the neurologists during medical examinations. Good results for correlation were achieved in several works (Pierleoni et al., 2014), which primarily used Pearson's coefficient (Salarian et al., 2007b; Cavallo et al., 2013; Kostikis et al., 2015; Rigas et al., 2016) and Artificial Neural Network (ANN) (Bazgir et al., 2015). Several machine learning approaches, including Support Vector Machine (SVM) classifier and Random Forest (RF), were implemented to predict the severity of tremor symptom (Kostikis et al., 2015). SVM (Hossen, 2012; Ghassemi et al., 2016; Surangsrirat et al., 2016), ANN (Hossen, 2012), combined Hidden Markov Model (HMM) (Rigas et al., 2012) Linear Discriminant Analysis (LDA), and *k*-means clustering (Ruonala et al., 2014a) were also used to distinguish between different groups of people (e.g., PwPD and HC, Parkinsonian and subjects with ET, or tremor PwPD and PwPD with other motor symptoms).

An alternative application was proposed by Hwang et al. (2009), who analyzed whether a light load can suppress tremor in the distal body segments. They demonstrated that in a PwPD the tremor is not suppressed, but actually it is enhanced in the proximal segments. They speculated that the application of greater inertial loads could reduce the tremor but could also be dangerous for patients that presented difficulties in balance and in postural strategies differently from healthy people. A load was also used by Ruonala et al. (2014a) who demonstrated that increasing the load to 1 or 2 kg decreased the accuracy in discrimination between PwPD, HC, and ET subjects.

From a different perspective, Fukumoto (2014) studied the effect of Ldopa treatment on tremor symptoms and found an increase of mean frequency and decrease of tremor power except for PwPD affected by motor fluctuations. Additionally, they found that visual and sound cues on tremor PwPD are able to improve tremor symptoms, similarly to the pharmacological therapy, although the Ldopa effect is most effective.

#### Recommendations and trends

According to Zhou et al. (2016), the harmonics of real tremor are not sinusoidal, as those studied in some works to simulate or control the PD tremor, but they vary over time. Thus, papers in which only tremor is simulated were not included in the review. For this reason, inclusion of a large number of PwPD who are significantly affected by tremor is critical to test the efficacy of the proposed systems in measuring the severity of the symptoms (Ghassemi et al., 2016). Indeed, even though some papers provided for the recognition of tremor severity, difficulties remain in distinguishing between adjacent levels to define the correct stage of pathology (Rigas et al., 2012). In addition, the discrimination between patients with similar symptoms but different pathologies (i.e., Parkinsonian tremor and ET) (Hossen, 2012; Hossen et al., 2013) is not easy to achieve, but it is crucial for a correct diagnosis and treatment of the disease (Hossen, 2012; Thanawattano et al., 2015; Ghassemi et al., 2016; Surangsrirat et al., 2016). For example, although ET patients have a tremor that is dominant during action and posture tasks, while PD patients particularly have tremor during rest, it is not a simple matter to find features that discriminate well between the two groups. However, power spectral density seemed to be a good measurement (Hossen, 2012; Pierleoni et al., 2014).

From a technical point of view, uniaxial accelerometers are not sufficient to adequately analyse the motion, whereas the use of triaxial inertial sensors can provide a more detailed investigation regarding tremor detection. Tremors in hands vary from one person to another and may occur more in specific axes rather than others (Salarian et al., 2007b; Alhamid et al., 2010; Thanawattano et al., 2015). Also, the integration of IMU with other typos of sensors, such as EMG, can improve the accuracy (Hossen et al., 2010; Hossen, 2012; Ruonala et al., 2014a,b; Kwon et al., 2016; Mailankody et al., 2016) and the range of the measurements, so additional different typologies of sensors could be investigated with the goal of improving tremor recognition. Since the intra-individual variability of RT and PT frequency in the dexterity-dominant lower limb was lower in PwPD than in HC, and RT frequency differed between upper and lower limbs in PD, devices able to identify minute variations which are imperceptible upon expert clinical exam can be used to differentiate a diseased person from a healthy one (Scanlon et al., 2013).

Regarding the wearability of the devices, the use of gloves in which to insert the sensors does not seem to be an optimal solution because of the disadvantages due to the noises caused by the electronic parts and the discomfort related to the device, which is not adaptable to different hand sizes. On this topic, the solution proposed by Cavallo et al. (2013) seems to be promising in terms of wearability, portability, light weight, performance, and ease of use. In addition, the wrist-worn Parkinson's Kinetigraph System can accurately detect tremor over an extended time (Braybrook et al., 2016). Also, smartphone-based solutions (Daneault et al., 2012; Bazgir et al., 2015; Kostikis et al., 2015) could be an alternative for the measurement of tremor, even if the need to have a custom-made glove-case makes the device usable for only a short time. Additionally, the RT is identified consistently, whereas the measured PT correlates weakly with the clinical assessment, likely because the mass of the smartphone affected the dynamics of the hand/arm system (Kostikis et al., 2015). Added benefits of smartphone use are the common availability and the fact that smartphones do not require downloads or memory-consuming installation because the service provided is web-based. Further, the use of smartphones can provide for a ubiquitous assessment of the disease both in the clinical setting and the home environment. Finally, smart clothes can represent an additional solution that is comfortable to wear and records data independently from a laboratory or technical staff for long-term monitoring (Niazmand et al., 2011).

The systems must be portable and lightweight to avoid disturbing the characteristics of the tremor; capable of being mounted to a predetermined anatomical anchor point (Alhamid et al., 2010; Cavallo et al., 2013); and able to provide timely feedback to the users. Thus, a wired system (i.e., connected by USB cable) to implement an offline analysis or a prototype of large dimensions (Salarian et al., 2007b) must be overcome in favor of totally wireless devices equipped with algorithms for real-time data analysis able to process the tremor quantification and prediction models. For this purpose, dynamic algorithms and models that allow the examination of the time-varying nature of tremors (Rigas et al., 2012) in the presence of unscripted and unconstrained voluntary movements (Cole et al., 2011; Roy et al., 2011) could be a valid solution. Tremor suppression is another important problem to improve QoL in PwPD who suffer with this symptom, and devices able to accomplish this must be investigated and implemented. For this purpose, Zhou et al. (2016) obtained crucial information in their recent study. They affirmed that PD tremor is composed of multiple harmonics with time-varying amplitude; thus, it is not a mono-frequency vibration. In particular, the 2nd and the 3rd harmonics are so strong that they cannot be neglected. Ignoring these components could lead to development of inefficient tremor-suppression devices. This phenomenon of harmonic peaks in higher-amplitude tremors carries also to differential diagnostic information when different typos of tremors must be recognized (e.g., Parkinsonian tremor and ET) (Hossen et al., 2010).

### Application 3: Body motion analysis

The cardinal features of PD are tremor, postural instability, muscular rigidity, and bradykinesia and/or hypokinesia. Thus, PD patients are characterized by a worsening of the motor performance that can be very disabling for them. These symptoms appear evident in different parts of the body, such as trunk, and lower and upper limbs. Generally, the symptoms are assessed by the neurologist during medical examination through visual inspection, in which the patients are asked to perform typical tasks described in the motor section of MDS-UPDRS (MDS-UPDRS III). In particular, for lower limbs the most investigated tasks are gait, including the disabling common complication known as freezing of gait (FOG), and the Timed Up and Go (TUG) test. For upper limbs, the research focused on finger tapping, alternating hand movement, pronation/supination, and finger-to-nose movement. Only one work concerning a multimodal system able to analyse motor tasks from both upper and lower limbs was found (Oung et al., 2015). Totally, 90 papers were assessed within this application. Considering the wide range of impairments related to the body motion, this application area is divided into five sub-sections, concerning different body segments or symptoms, which are named: gait and TUG test, freezing of gait, postural instability, upper limbs and other symptoms (leg agility, rigidity, and arms swing).

#### Gait and timed up and go (TUG) test

Gait is the most examined task in the studies for the analysis of motor performance in PwPD (37 papers were included). Motion capture systems (e.g., ultrasound system, optical system, and/or forceplates) are the gold standard for motion analysis. These systems are typically used to assess the parameters characterizing gait, but they are expensive, unportable, and usable only in laboratory environments. Recent studies also support the use of IMUs to assess objectively the movement of PwPD by demonstrating the validity of IMUs in comparison to motion capture systems (Del Din et al., 2016; Ferrari et al., 2016; Sejdić et al., 2016). Several studies showed the use of accelerometers (Stamatakis et al., 2011; Palmerini et al., 2013; Jarchi et al., 2015; Del Din et al., 2016; Sejdić et al., 2016), gyroscopes (Fatmehsari and Bahrami, 2010; Grimpampi et al., 2013), or both methods (Oung et al., 2015; Trojaniello et al., 2015; Ferrari et al., 2016), placed on different segments of the body (e.g., shank, thigh, foot, lower back) to measure the performance of gait in PD patients, in particular to assess both TUG test and long-distance walking, to distinguish between HC and PwPD during specific tasks (Esser et al., 2013; Mariani et al., 2013; Del Din et al., 2016; Table 3). An alternate approach foresees the use of a smartphone-equipped triaxial accelerometer (Arora et al., 2014) or a StepWatch worn on the wrist (Schmidt et al., 2011) to capture the movement of patients during preset gait tests. Statistical (e.g., mean, variance, skewness, kurtosis), frequency (e.g., energy, power spectral density, fundamental frequency), and spatiotemporal/kinematic (e.g., stride length, TUG time, stride velocity) features were extracted and analyzed. Step or stride segmentation were key points for the gait analysis to recognize heel-strike and toe-off times characterizing the gait cycle and the complete walk (Barth et al., 2013; Del Din et al., 2016; Parisi et al., 2016). The experimental protocols were principally based on TUG exercise and gait. The TUG test consisted of standing up from the chair and walking a 3 m (or 7 m) distance at a normal speed, followed by a turn of 180° and walking back, and ending with another turn of 180° and sitting down on the chair (Salarian et al., 2010; Weiss et al., 2010; Al-Jawad et al., 2012; Mariani et al., 2013; Palmerini et al., 2013; Reinfelder et al., 2015). Restricted sit-to-stand (Si2St) task with feet fixed on the floor without any linear translation movement (Giuberti et al., 2015); extended TUG test (ETUG) with 10 m to walk and wide curve trajectory (Caldara et al., 2014); and Instrumented Stand and Walk Test (ISAW), which is a TUG in which the phase of standing up and sitting down are not included (Curtze et al., 2016; Horak et al., 2016), are variations on the traditional tasks. Alternatively, other works focused on gait tests on short (Esser et al., 2013), moderate (Schmidt et al., 2011; Arora et al., 2014), and long distance (Weiss et al., 2011), including 180° turns (Mariani et al., 2013; Rahimi et al., 2014); or straight walking at different speeds (e.g., comfortable, slow, fast) (Salarian et al., 2013; Del Din et al., 2016); or random walking with initiated stops and several 360° turns; or basic mobility-related activities (e.g., lying, standing) and domestic activities (Barth et al., 2013; Yoneyama et al., 2016). Only Barth et al. (2011) and Oung et al. (2015) proposed to analyse exercises able to assess foot mobility (e.g., heel-toe tapping or foot rotation), whereas, Parisi et al. (2015) proposed a comparative outlook of different tasks: gait, sit-to-stand, and leg agility. Others (Lord et al., 2010; Rochester et al., 2010) implemented experimental protocols which include single, dual, and multiple tasks to analyse the effect of external cues on gait strategies. Only one work (Salarian et al., 2009) was focused on turning; it recognized differences between early PwPD and HC with excellent sensitivity and reliability thanks to the automatic detection of all turns. The majority of the works compared the performances of a group composed of PwPD and a group of control subjects (Barth et al., 2013; Palmerini et al., 2013; Arora et al., 2014; Oung et al., 2015; Parisi et al., 2015), and showed that the second group had better results in terms of time of execution, speed (Horak et al., 2016), regularity, cadence, symmetry, stride length (Demonceau et al., 2015), amplitude, and slope (Weiss et al., 2011). Others implemented multi-class classification to distinguish among HC, PwPD without gait disturbance, and PwPD with gait disturbance (Tien et al., 2010) or compared the performance of HC, PwPD, and subjects with dementia (Yoneyama et al., 2016). Moreover, recent European research projects, including REMPARK (Cabestany et al., 2013), PERFORM (Cancela et al., 2011), and CuPiD (Ferrari et al., 2016), used systems based on wearable IMUs to examine disease management and assessment with artificial intelligence and to try to identify the gait and movement of PD disorders.

**Table 3:** Papers about gait and TUG analysis.

**References**	**Tech**.	**Sensors place**	**Rec. freq**	**Experimental design**	**Subjects**	**Feature extracted**	**Analysis/classifiers**	**Classifier performance or findings**
Del Din et al., 2016	ACC	Lower back (L5)	50 Hz	10-m walkway	30 PwPD, 30 HC	Mean, SD, variability and asymmetry of stride, stance and swing time, step length, step velocity	ICC; Pearson correlation; *t*-Test	ICC > 0.9 for mean step time, stance time, step length both for HC and PwPD. ICC > 0.9 for step velocity for HC. Significant difference in step variability between PwPD/HC
Ferrari et al., 2016	ACC, GYRO	Feet	100 Hz	1st study: walking over a treadmill 1.8, 2.7, 3.5 km/h (1 min). 2nd study: walking in a straight line	1st: 11 HC. 2nd: 16 PwPD	Strides number (#), stride length, stride time, stride velocity	ICC; Zero velocity update algorithm, RMS	Study 1: 4.0% RMS of the differences normalized to the mean stride length. Study 2: 2.9% RMS of the differences in % of the mean stride length. ICC > 0.9
Sejdić et al., 2016	ACC	Lower back (L3)	100 Hz	Gait on treadmill: 3 min walking at preferred pace, rested, 3 min walking at a slower speed (−10% from preferred speed)	10 PwPD, 14 HC, 11 patients with neuropathy	Swing and stance time, single and double support (DS) time, HRs of the trunk ACC	*t*-Test; ANOVA; Mixed models	Differences in: group (*p* = 0.04) and speed (*p* = 0.02) for HRs for ACC/motion capture comparison. Magnitudes of HRs 5–10% lower in ACC than motion capture system.
Palmerini et al., 2013	ACC	Lower back	100 Hz	iTUG test 7 m	20 PwPD, 20 HC	Time of: TUG, sit-to-walk, gait, turning, walk-to-sit. RMS of: sit-to-walk, gait, turning, walk-to-sit. Normalized jerk score of the ACC of: sit-to-walk, gait, turning, walk-to-sit. Step time, SD of step time, CV of step time. Phase of step, SD of phase, CV of phase. Phase coordination index (PCI) during gait. HRs of the trunk ACC	ANOVA; ICC; Pearson correlation; LDA, Quadratic discriminant analysis (QDA), Mahalanobis (MC) classifiers	Misclassification rate: 22.5% for LDA, 27.5% for QDA, 37.5% for MC. Correlation of: (i) time TUG with gait and posture subscore (*r* = 0.6, *p* = 0.005), rigidity subscore (*r* = 0.46, *p* = 0.04) and PIGD subscore (*r* = 0.51, *p* = 0.02); (ii) HRs with gait and posture subscore (*r* = −0.47, *p* = 0.037); (iii) turning RMS in the VT direction with gait and posture subscore (*r* = −0.58, *p* = 0.007) and rigidity subscore (*r* = −0.47, *p* = 0.038). ICC > 0.8 for time TUG but no group differences.
Grimpampi et al., 2013	ACC, GYRO	Waist	100 Hz	Walking at a self-selected speed along a 12 m rectilinear pathway	11 PwPD, 13 after stroke	Pitch, roll and yaw angles, walking speed	RMS and correlation coefficient	RMS < 1° for pitch and roll; RMS = 1.3° for yaw for IMU/optoelectronic system comparison. Coeff = 0.8 for correlation
Trojaniello et al., 2015	ACC, GYRO	Lower back (L4/S2)	128 Hz	Walking back / forth for 1 min along a 12 m walkway with the instrumented mat placed 2 m from the starting line (self-selected, comfortable speed)	10 PwPD, 10 HC, 10 hemiparetics, 10 Huntington disease	Gait cycles, mean and SD of stride time, stance time, swing time, step time, gait velocity	Wilcoxon signed-rank test, Friedman test	Stride time, step time, stance and swing duration errors for PwPD were significantly larger than HC
Mariani et al., 2013	ACC, GYRO	Feet	200 Hz	TUG (3 m) and gait at self-selected speed on moderate (2 × 20 m) and long (4 × 50 m) distance, including straight walking and 180° turns	10 PwPD, 10 HC	Stride velocity, stride length, turning angle, path length, swing width, inter-cycle variability	ICC; mean ± SD	acc. ± prec. of 2.8 ± 2.4 cm/s and 1.3 ± 3.0 cm for stride velocity and stride length estimation compared to optical system
Esser et al., 2013	ACC, GYRO	L4	100 Hz	10-m walkway free of obstacles, self-selected walking pace	14 PwPD, 10HC	Cadence, stride length, walking speed, parameters extracted by phase plot analysis (i.e., spread and width of the cloud data points)	ICC; *t*-Test; Phase plot variability analysis	ICC > 0.9; Difference (*p* = 0.041) for walking speed between PD/HC. The width of data point is affected by a change in step length, the spread in data point is affected by a change in cadence and step length
Schmidt et al., 2011	StepWatch Activity Monitor	Wrist	Not reported	15 m walking	20 PwPD and multiple sclerosis (MS)	Stride count	Pearson coefficient	*r* = 1.0 for PwPD correlation between SAM/GaitMat II. *r* = 0.99 for MS correlation between SAM/GaitMat II
Parisi et al., 2016	ACC, GYRO	Chest, thighs	102.4 Hz	Gait	34 PwPD	Stride time, stance time, DS time, step and stride length, limp, step velocity, thigh rom, cadence, step regularity, symmetry, spectrum power	PCA; NCC, kNN, SVM	Prec., sens. and spec.: 66.48, 31.83, and 88.03%
Salarian et al., 2010	ACC, GYRO	Sternum, forearms, thighs, shanks	200 Hz	TUG (3 m) and iTUG (7 m) including: gait, turning, Sit to Stand (Si2St), Turn-to-sit	12 PwPD (early), 12 HC	*Gait*: cadence, stance, DS, limp, ROM of shank, thigh and knee, stride length and velocity, peak swing velocity, arm swing pitch and yaw, peak arm swing velocity, arm swing speed asymmetry, peak trunk horizontal and sagittal velocity, ROM of trunk. *Turning*: peak angular velocity, duration, steps, average and max step time, # double steps. *Si2St*: peak and average angular velocity, duration, ROM of trunk. *Turn-to-sit*: duration, ROM of trunk, steps, average and max step time, # double steps	ICC; Wilcoxon rank-sum test	ICC > 0.9 for temporal measures except for limps; ICC = 0.75 for gait measures; ICC = 0.23 for Si2St measures; ICC = 0.67 for turning measures; ICC = 0.50 for turn-to-sit measures. Differences in cadence (*p* < 0.006), angular velocity of arm-swing (*p* < 0.005), turning duration (*p* < 0.023), and time to perform turn-to-sits (*p* < 0.023) between early PwPD/HC
Weiss et al., 2010	ACC, ECG, GSR, force sensors	Lower back (L3/L5)	256 Hz	TUG	17 PwPD, 15 HC	Mean and SD of: time of TUG, time of Si2St, time of stand-to-sit (St2Si), Range Si2St, Range St2Si, Jerk Si2St, Jerk St2Si. Median and SD of ACC	*t*-Test; Pearson correlation	TUG duration (*p* < 0.02), median ACC (*p* = 0.02), SD ACC (*p* < 0.004) higher in PwPD than HC. Range and jerk of ACC lower in PwPD than HC (*p* < 0.006). Jerk Si2St correlated with UPDRS (*r* = 0.56; *p* = 0.02) and HY scores (*r* = 0.49, *p* = 0.04)
Giuberti et al., 2015	ACC, GYRO	Chest	102.4 Hz	Si2St	24 PwPD	Forwards/backwards/total duration, forwards/backwards/average bending amplitude and bending speed	PCA; NCC, kNN, SVM	Miscalssification rates: 3.7% for UPDRS = 0; 100% for UPDRS = 0.5; 50% for UPDRS = 1; 71.4% for UPDRS = 2; 100% for UPDRS = 2.5; 100% for UPDRS = 3. No data for UPDRS = 2, 3.5, 4
Curtze et al., 2016	ACC, GYRO	Wrists, ankles, lumbar segment, sternum	Not reported	ISAW: to stand still for 30 s, initiate gait with the most affected leg, walk 7 m at comfortable pace, turn 180°, and walk back to the starting location (3 times)	104 PwPD	34 measures of gait and balance into 6 domains: postural sway, initiation of gait, gait arm and trunk movement, gait dynamic stability, turning	Spearman correlation; false discovery rate correction	30 significant associations between gait and balance measures and clinical scales. Turning and gait–pace are most indicative of patient status
Horak et al., 2016					100 PwPD, 21 HC	90 measures of gait and balance into 6 domains: sway area, sway freq, gait speed, gait trunk, gait timing, arm asymmetry	ICC; Pearson correlation; *t*-test	ICC > 0.75 for 30 features. Gait and postural sway measures not highly correlated. *r* = −0.62 between gait trunk domain and PIGD subscale. Largest differences PD-ON/HC in gait speed and gait trunk
Weiss et al., 2011	ACC (Mobi8, TMSI)	Lower back	256 Hz	Validation study: 1 min, straight-line walk at a self-selected, comfortable pace inside a long hallway. Gait test: straight-line walk (~25 m × 2). ADL simulation: 500 m walk at comfortable, self-selected speed	22 PwPD, 17 HC	Stride time, stride time variability (validation study only). Dominant freq, amplitude, width (FD), and slope of the main freq of the PSD in the 0.5–to 3.0-Hz band	*t*-tests 2-tailed; paired *t*-tests; Pearson coefficients	Width larger, and amplitude and slope smaller in PwPD compared to HC [validation study and ADL simulation (*p* < 0.02)]. Width decreased, and amplitude and slope increased with anti-Parkinsonian medications (*p* < 0.007). Significant correlations ACC-derived measures/UPDRS-Gait5
Rahimi et al., 2014	FAB system BioSyn® (ACC, GYRO)	Head, arm, forearm, trunk, pelvis, thigh, shank	100 Hz	Walking, walking turns of 180° and fast walking (3 trials)	11 PwPD	Mean and peak amplitude values of each of 59 joint variable	Change space; Least Absolute Shrinkage Selection Operator (LASSO)	Correctly predicted 5 cases of improvement and 2 cases of worsening after medication
Salarian et al., 2013	GYRO	Shanks	200 Hz	Walking down a 20 m straight hallway both with comfortable speed and fast speed	10 PwPD, 10 HC	Joint angle kinematics including flexion/extension angles at the hip, knee, ankle joints, cadence, % of swing, stance, DS phases	Linear mixed model	Double pendulum model with 2 GYRO on shanks reduces the number of sensing units compared to more complex methods, with relatively small impact on accuracy
Yoneyama et al., 2016	Portable rhythmo-gram (ACC)	Waist	100 Hz	ADL in the community for 24 h with the device attached at all times (including sleeping hours) except when changing clothes or taking a bath	13 mild and 13 severe PwPD, 13 HC, 13 mild and 13 severe MCI/dementia	Features based on: gait ACC, gait variability, gait cycle, number of gait data	Kruskal-Wallis (KW) test with *post hoc* Steel-Dwass test	The proposed gait measures may deserve to be used for the quantification of disease-specific context-dependent aspects. Gait variability is the lowest in mild PwPD
Parisi et al., 2015	ACC, GYRO	Chest, thighs	102.4 Hz	Comparative investigation of: Leg agility (LA), Si2St, Gait	34 PwPD	*LA*: angular amplitude and speed, pause, regularity, repetition freq, thigh inclination, angular velocity power spectrum. *Si2St*: forwards/backwards/total duration, forwards/backwards/average bending amplitude and bending speed. *Gait*: stride, stance time and DS time, step and stride length, limp, step velocity, thigh ROM, cadence, step regularity, symmetry, spectrum power	PCA; NCC, kNN and SVM	Prec., sens. and spec.: 34.55, 25.17, 84.52% in LA task; 28.00, 25.63, and 77.51 in Si2St task; 66.48, 31.83, and 88.03% in Gait task
Rochester et al., 2010	ACC, biofeedback	Legs, sternum	25 Hz	Single task, dual task, retention of single and dual task over 3 weeks	76 early and 77 late PwPD	Walking speed, step length, step freq	Multiple linear regression models; 2-tailed analysis	Significant training effect for: Single task in speed with cues, step length with/without cues, cadence without cue; Dual task in speed and step length with/without cues. No significant retention effect
Lord et al., 2010	ACC (Vitaport Activity Monitor)	Not specified	Not reported	(i) Single task: standing up from a chair and walking to the kitchen; (ii) dual motor task: specific ADL; (iii) cognitive task; (iv) multi-task.	29 PwPD	Gait speed, interference effect	Moment correlation coefficients, linear regression	For gait speed: within-subject effect during the functional walk (*p* < 0.001), with gait speed slower during dual and multi-task performance. Higher UPDRS-III scores resulted in a significantly slower walking speed for all conditions. Participants with impaired sustained attention walked more slowly in single and dual motor conditions.
Demonceau et al., 2015	ACC	Lower back (L3/L4)	100 Hz	Walking at the self-selected pace along a 36-m-long track in a wide, clear, and straight hallway	32 PwPD (HY < 2), 32 PwPD (HY = 2/3), 32 HC	Stride length, cadence, regularity index, symmetry index, walking speed and mechanical powers yielded in the cranial-caudal, AP and ML directions	ANOVA, KW, Tukey, Mann- Witney, Wilcoxon, Correlation coefficients; Multivariate regression, ORs	Difference between PwPD groups in regularity index (*p* = 0.009, OR = 0.98). Significant difference between PwPD groups and HC in symmetry index, speed, stride length, mechanical powers in ML and AP directions. Significant (*p* < 0.05) but low (*r* < 0.5) correlation with clinical data. Regularity index and power in ML direction discriminated the 3 groups.
Yoneyama et al., 2013	ACC	Waist	100 Hz	Test 1: walking 200 steps (9 dictated paces). Test 2: stepping on the same spot and intentional side-to-side body sway; normal walking with the device to the left side of the body; walking asymmetrically; jumping forward with both legs (each 10 s). Test 3: collecting ACC data in hospital (10 min) and outside (24 h)	Test 1: 11 HC; Test 2: 1 HC; Test 3: 12 PwPD	Gait cycle, average VT ACC per cycle	Threshold levels criterion	Test 1: 97.2% sens., 97.4% spec., 97.3% acc. Test 2: 100% sens., 100% spec., 100% acc. Test 3: 94.0% sens., 95.7% spec., 95.4% acc.

##### Recommendations and trends

As with other applications previously analyzed, some works presented limited datasets, investigating groups that were not age-matched (Ferrari et al., 2016) and sometimes including other pathologies in addition to PD (Schmidt et al., 2011; Salarian et al., 2013). Thus, bigger sample sizes are needed to confirm the significance of the novel gait parameters (Mariani et al., 2013). In several studies, moderate patients (e.g., HY = 2/3, Palmerini et al., 2013; Yoneyama et al., 2013; Sejdić et al., 2016) were involved, so the difference in performance between PwPD and HC are easily identifiable. The recruitment of PwPD in the first stage of the disease (i.e., HY = 1) should be primarily investigated to demonstrate the accuracy and the objectivity of the technological solutions with respect to the traditional clinical evaluations (Demonceau et al., 2015), aiming to achieve early diagnosis of the pathology (Barth et al., 2011). Many gait analysis protocols have been developed to complete the medical exam of PD patients, but the optimal method remains under debate (Demonceau et al., 2015). Regardless, the use of inertial sensors placed on different parts of the body seems to be a promising method for objective estimation of the parameters of the gait (Barth et al., 2013; Del Din et al., 2016; Ferrari et al., 2016). The performance of the inertial sensors is different from that of commercial pedometers that are less accurate in quantifying gait performances. Although the TUG test, which includes turning movements, was analyzed in several papers, and the importance to classify the different phases with the TUG test is recognized (Reinfelder et al., 2015), only one work specifically dealt with the rotation task. This is likely because turning is not directly measured in UPDRS, and the PIGD sub-score as a clinical measure of reference has limited compliance (Salarian et al., 2009). The majority of the papers aimed to distinguish between the PwPD and HC and compared the spatiotemporal and frequency features measured during the protocol adopted. Only a restricted number of studies (e.g., Salarian et al., 2010, 2013; Tien et al., 2010; Parisi et al., 2015) focused on developing full biomechanics analysis to measure biomechanical parameters, such as joint range of motion, ankle dorsiflexion, finger flexion, etc., and investigated the benefit of using such kind of features in artificial intelligence algorithms. Within the same papers, different feature selection methods could be examined and compared; those revealing the best accuracy in distinguishing between the two groups were selected [e.g., SVM, LDA, RF, odds ratios (ORs), k nearest neighbors (kNN), nearest centroid classifiers (NCC), *t*-test]. Parisi et al. (2015) also reported good results in correlating kinematic features and UPDRS scores, although the automatic system tends to underestimate the actual UPDRS scores. The lack of a meaningful correlation might be due to the relatively blunt nature of UPDRS in assessing PD symptoms (Yoneyama et al., 2013). Alternatively, Barth et al. (2013) implemented a step segmentation algorithm based on Dynamic Time Warping (DTW), which has the main advantage that the two input series do not need to be aligned in the time domain, and the error caused by the non-linear relation of the two series can be avoided. Other papers pointed out conflicting results, even when starting from identical hypotheses (Salarian et al., 2009; Palmerini et al., 2013). For Palmerini et al. (2013), in fact, temporal measures of PwPD are normal compared to HC, but patients are characterized by reduced smoothness and dynamics in trunk movement during gait and turining. However, the misclassification rate of 22.5% in the early-mild stage of the disease is high. Further, the separation between PwPD and HC is not challenge anymore, because an accurate distinction within the group of PwPD should be achieved to assess the severity of the pathology at each moment (Barth et al., 2011). This task remains difficult, even though significant differences between mild and severe PwPD (Yoneyama et al., 2016), as well as between mild and moderate PwPD (Demonceau et al., 2015) were seen. Further, Parisi et al. (2015) revealed the contribution of the sit-to-stand task to distinguish between patients with slight and mild symptoms and those who manifest moderate or severe impairments. Finally, for the TUG test, accelerometer-derived parameters, in addition to test duration, could represent complementary and objective biomarkers of PD to assess the pathology progression and therapeutic response (Weiss et al., 2010). To minimize the invasiveness of the devices and to improve the acceptability of the systems proposed, it is important to try to reduce the number of sensor units using existing biomechanical models and place the devices in a way that does not interfere with gait (Salarian et al., 2013). Fatmehsari and Bahrami (2010), for example, demonstrated that a single gyroscope attached to either shank or thigh is sufficient to discriminate between PwPD and HC by calculating non-linear features. Trojaniello et al. (2015) confirmed that a single IMU placed on the lower back works well for healthy subjects, but it shows difficulties for impaired gait. This result was confirmed by Ferrari et al. (2016), who found that shuffling gait could mask the proper detection of initial contacts and foot-off events. In contrast, Sejdić et al. (2016) obtained good results in pathological subjects, as did Del Din et al. (2016), who affirmed that a single accelerometer on the lower back is sufficient for measuring gait characteristic, including asymmetry and variability. Therefore, in pathological situations, the use of sensors placed on both legs is recommended (Reinfelder et al., 2015) so that data from left and right sides can be merged for the final evaluation (Ferrari et al., 2016). A smartphone-based solution (Arora et al., 2014) or the StepWatch (Schmidt et al., 2011) can be innovative in terms of wearability because the sensors are hidden in common tools, and they can accurately count the strides. However, these solutions do not allow measurement of clinical features of interest such as stride length, so a complete analysis of the movement is not possible, and direct comparison with other systems is not feasible. For future implementations, it is crucial that the results of the gait analysis are shown immediately after execution of the test, through the development of semi-automated operations (Caldara et al., 2014) or dedicated applications available on smartphones (Ferrari et al., 2016), to enable real-time gait analysis. The algorithms should automatically detect all transitions and all turns, showing differences between HC and PwPD and good test-retest reliability (Salarian et al., 2010), even if large variations in results are common due to different walking styles. Although the principal aim of gait analysis is to quantify the motor performance of the patients to provide a more accurate diagnosis of the pathology, gait analysis can be associated with other applications, including rehabilitation, supporting decision-making (Grimpampi et al., 2013), biofeedback for gait monitoring, and fall prevention (Caldara et al., 2014). The use of adequate external cues can improve the gait stability for early/mild patients, but the cues become less effective for advanced patients. However, the use of auditory, visual, and somatosensory cues during single and dual tasks enhance motor learning in PwPD (Rochester et al., 2010), so they could reasonably support rehabilitation programs. Differently, the implementation of dual and multi tasks that measured selective, divided, and sustained attention, negatively interfered with the gait (Lord et al., 2010).

#### Freezing of gait

FOG is one of the more disabling complications, especially in elderly long-term, advanced PwPD. Motor blocks are a subtype of the FOG phenomenon that primarily affect the gait initiation process. They include delayed release of anticipatory postural adjustments (APA), hypokinetic APA (reduced scaling), and bradykinetic APA (abnormal timing), suggesting the existence of a pathophysiological mechanism that involves both locomotor networks and cortical areas (Delval et al., 2014). FOG episodes mainly appear at the gait initiation, when the patient must turn or when to the patient must pass through narrow spaces. The gold standard for FOG evaluation is direct or video recorded gait—even if, often, the FOG questionnaire (FOG-Q) is administered (Bächlin et al., 2009). Worsening coordination during gait is another feature characterizing PwPD, and is directly correlated to FOG severity (Mazilu et al., 2016). In the 20 studies included in this review, IMUs were used alone, with other sensors, or integrated in different technological devices to improve the detection of FOG events (Table 4). Force sensors (Djurić-Jovičić et al., 2014b), EMG (Cole et al., 2011), headsets (Lorenzi et al., 2015), earphones (Bächlin et al., 2009, 2010), ECG and Galvanic Skin Response (GSR) sensors (Mazilu et al., 2015), and a portable four-channel wireless electroencephalogram (EEG) system (Handojoseno et al., 2012, 2013, 2014, 2015) were the most common supplementary devices used to provide biofeedback. In contrast, Morris et al. (2013) proposed a validated method to assess the phenomenon using a computer-generated animation and reconstructed data coming from IMUs. Capecci et al. (2016) used a smartphone at the hip joint to record gait data to detect FOG events. Mazilu et al. (2016) proposed to apply IMU on the wrist since movement on the upper limbs is also highly correlated with FOG events, and the wrist seems to be a convenient place in terms of unobtrusiveness, usability, and acceptability. Furthermore, results from both ankles and wrist are minimally better than those obtained by ankles only. To define a parameter to assess FOG episodes, Moore et al. (2008) analyzed the frequency characteristics of vertical leg movement during walking. They introduced the Freeze Index (FI), the ratio between the power of the gait signal in the “freeze” band (3–8 Hz) and the power in the “locomotor” band (0.5–3 Hz). When experiencing a freezing episode, a “trembling” of the leg was observed, reflected in the power spectra of vertical leg movement with high-frequency components in the band 2–6 Hz. Zach et al. (2015) also adopted the FI to identify freezing episodes by analyzing FOG-eliciting tasks as rapid full turns and walking with rapid short steps. They obtained low specificity due to false positive events detected by the sensor but not revealed by video analysis. Additional information about step cadence can improve sensitivity and specificity for FOG event recognition, avoiding false event detection (Capecci et al., 2016). Tripoliti et al. (2013) instead proposed to measure the entropy related to the freezing event, since it is a nonlinear parameter, as is the FOG disorder. Djurić-Jovičić et al. (2014b) proposed a novel method that used the Pearson's correlation coefficient to define the “representative stride” and the “normal zone” to separate normal from abnormal gait, distinguishing also between straight and turning strides. Alternatively, Cole et al. (2011) proposed a dynamic neural network to better capture the time-varying nature of FOG, because the method enabled them to learn how the features representative of FOG events can change over time. Differently, Handojoseno et al. performed frequency and wavelet analysis to extract significant features from EEG signals and then used MLPNN to detect FOG episodes with accuracy ranging between 70 and 80% (Handojoseno et al., 2012, 2013, 2014, 2015). Vibration and auditory biofeedback methods were implemented (Bächlin et al., 2009, 2010; Mazilu et al., 2014) to provide a cue to alert the patient about the occurrence of a FOG event. This kind of intervention can be helpful in preventing falls due to FOG episodes, with the aim to reduce major complications for the PwPD both in terms of health and costs. To allow the biofeedback intervention and the alert to the patient, a real-time processing of data is needed (Bächlin et al., 2009; Mazilu and Hardegger, 2012; Mazilu et al., 2014). Recently, in European projects (e.g., REMPARK, CUPID), systems for PD that include the detection and intervention of FOG episodes (Cabestany et al., 2013; Mazilu et al., 2014, 2015, 2016), propose devices such as GaitAssist, and are equipped with two IMUs and a smartphone for active support of gait initiation, turns, and response inhibition, were developed (Mazilu et al., 2014).

**Table 4:** Papers about FOG analysis.

**References**	**Tech**.	**Sensors place**	**Rec. freq**	**Experimental design**	**Subjects**	**Feature extracted**	**Analysis/classifiers**	**Classifier performance or findings**
Mazilu et al., 2016	ACC, GYRO	Wrists, ankles	128 Hz	Walking: straight line, 8-shape, random turns and changes in direction, the Ziegler protocol (with/without cognitive tasks). Real-life walking session (hospital): walking randomly with voluntary stops, changes of direction, using the elevator. Non-walking sessions: rest periods, sitting, standing	18 PwPD (11 with FOG during the study), ON state	Mean and SD from ACC and GYRO, power in different freq band	Mutual Information; Pearson correlation; ANOVA; Hit rate, false positive events, spec. and detection latency	# false positives increases by 40% when using wrist movements, compared with ankle data. Data from both ankles and one wrist improve only by 0.03 in spec., compared with ankle. 0.94 hit rate and 0.66–0.8 spec. for FOG detection events
Djurić-Jovičić et al., 2014b	ACC, GYRO, force sensitive resistors	Shanks; force sensors into the shoe	100 Hz	To stand up from the chair and start walking including straight path, turns, U-turn, pass (very narrow) doors, and sit down (4 times)	12 PwPD, OFF state	Stride duration, spectrum analysis, shank orientation and displacement	Pearson correlation coefficients	Correct classification rate: 94.78% normal strides; 84.34% short strides; 78.13% very short strides; 100% FOG with trembling; 100% FOG with complete motor block; 87.80% shortening strides while turning
Lorenzi et al., 2015	ACC, GYRO, Headset	Shanks /Head	60 Hz	Walking some steps, passing through an open door, turning and going back	10 PwPD	VT ACC, K index	ANN (2 layers)	94.5% sens., 96.7% spec. 93.8% prec. and 95.6% acc. for FOG events detection
Bächlin et al., 2010	ACC, earphones	Thigh, shank, lower back	256 Hz	(i) Walking back/forth in a straight line, including several 180° turns; (ii) random walking in a reception hall space, including a series of initiated stops and >6 several 360° turns. (iii) walking simulating ADL included entering/leaving rooms, walking to the lab kitchen, getting something to drink, returning to the starting room with the cup of water. 1st session with Rhythmical Auditory System feedback, 2nd session without it. 10–15 min each session	10 PwPD (8 with FOG during the study), (8 OFF state, 2 ON state)	FI, PSD	N/A (Comparison to video analysis)	73.1% sens., 81.6% spec. for online FOG detection
Mazilu et al., 2015	ACC, GYRO	Wrists	128 Hz	Walking protocols in lab setting designed to provoke FoG including: walking with 360° and 180° turns, walking in straight lines and passing narrow corridors, or walking across the crowded hospital halls	18 PwPD (11 with FOG during the study), ON state	Mean and SD from ACC and GYRO, power in different freq band	C4.5 leave-one-out cross validation. Hit rate, false positive events, specificity, detection latency	Subject-dependent: 0.9 hit rate, 0.83 spec., average detection-latency of 1.53 s. Subject-independent: 0.9 hit rate, 0.7 spec., average detection-latency of 0.98 s.
Handojoseno et al., 2015	4 channel wireless EEG system	Head	500 Hz	Structured series of video-recorded TUG tasks The features were measured during normal walking, FOG onset, FOG	10 PwPD with significant FOG	PSD, Wavelet energy WE (α, β, γ, δ, θ), WE entropy and other freq features	Wilcoxon sum-rank test. MLPNN	Abnormal EEG hyperconnectivity involving frontal, occipital, parietal and central regions during transition to FOG and FOG episodes. 86.0% sens., 74.4% spec., 80.2% acc. for FOG detection
Morris et al., 2013	ACC, GYRO, Computer generated animation	Thighs, shanks, feet and lower back	50 Hz	TUG (5 m)	10 PwPD, OFF state	# FOG events, % time frozen	ICC; *t*-Test; ANOVA	FOG events: ICC = 0.35 from animation and ICC = 0.63 from video. % time frozen: ICC = 0.65 from animation and ICC = 0.73 from video. Observers scored more FOG episodes from animation relative to video (*p* < 0.0001). *p* < 0.001 between subjects for both % time frozen and # freezes.
Capecci et al., 2016	Smart phone with ACC	Hip joint	100 Hz	(i) TUG task (5 m). (ii) Cognitive TUG with dual task. (iii) Manual TUG carrying 2 full cups of water on a tray	20 PwPD (16 with FOG during the study), ON state	Step cadence, FI, PSD, EI (sum of locomotor and freeze bands)	ROC; McNemar's test. ICC; KW test, Chi Square test	ICC > 0.80 concerning FOG duration. PwPD performed the standard TUG faster than the Cognitive (*p* = 0.01) and Manual (*p* = 0.01) TUG tests. FOG events detection (acc.): 81.7% in Algorithm 1; 84.4% in Algorithm 2
Moore et al., 2008	ACC	Shank	100 Hz	Walking without assistance at a self-determined pace straight line, with at least 2 180° turns, to negotiate a narrow doorway and 3 obstacles. Subjects stood for 10 s at the end of each walking trial. Patients repeated the walking task over a 90 min epoch post-administration. Distance walked ranging from 1 to 90 m when “off” and from 35 to 94 m in the “on” state	11 PwPD with FOG, OFF state	FI and power in different freq band	N/A (comparison to video analysis)	Sens. in FOG detection with global threshold (78%) and individual thresholds (89%)
Zach et al., 2015	ACC	Lower back	100 Hz	(i) Normal walking (10 m, self-selected speed and stride length); (ii) walking rapidly (10 m); (iii) walking with short steps (10 m; self-selected speed); (iv) walking with short rapid steps (10 m); (v) rapid full turns: at least 4 turns in each direction	23 PwPD with FOG (16 with FOG during the study), OFF state	FI, freq spectra	Cohen's kappa; ROC	Cohen's kappa = 0.94. FOG events detection: 78% sens., 59% spec during full rapid turns; 64% sens., 69% spec. during walking with short rapid steps; 75% sens., 76% spec. overall
Tripoliti et al., 2013	ACC, GYRO	Wrists, ankle, waist, chest	50–60 Hz	(i) Lying on bed (5 min), (ii) rising from bed and sitting on a chair (5 min), (iii) standing up from the chair and performing ADLs (8 min): (1) walking 5 m, (2) opening/closing the door, (3) opening the door and straight walking 10 m, 4) walking back, (5) making a stop and drink from a glass of water, (6) walking back to the chair	5 PwPD with FOG; 6 PwPD without FOG; 5 HC, OFF state	Entropy	Naïve Bayes, RF, DT, Random Tree	81.94% sens., 98.74% spec., 96.11% acc. and 98.6% AUC using RF
Morris et al., 2012	ACC	Shanks	50 Hz	TUG (5 m)	10 PwPD, OFF state	# FOG events, % time frozen	ICC	% time frozen ICC: 0.73 inter-rater, 0.71 intra-rater, 0.93 all raters. # FOG ICC: 0.63 inter-rater, 0.44 intra-rater, 0.78 all raters

##### Recommendations and trends

As with the other applications, the number of patients involved in these studies is not very high. Furthermore, not all the patients experienced FOG during experimental phases, so the datasets are further reduced. To avoid this issue, the systems also could be tested at home, at convenient times during the day, to capture a higher number of FOG events. However, the implementation of a long experimental protocol that includes cognitive and physical dual-tasks seemed to be able to trigger FOG episodes also in the laboratory setting (Capecci et al., 2016). Nevertheless the major difficulty for FOG detection is its unpredictability, so it would be preferable to test the wearable sensors in everyday situation and not while performing structured test (i.e., TUG test). Moreover, the large variability between clinicians suggests that caution should be used when comparing subjective ratings across centers (Morris et al., 2012). The majority of the works implemented experimental protocols that included all the part of freezing (i.e., starting, turning and narrow) while only some works (Handojoseno et al., 2012, 2013, 2014, 2015; Morris et al., 2012, 2013; Zach et al., 2015; Capecci et al., 2016) applied TUG test for FOG detection, which is a test that not include “narrow” tasks. Generally the number and the duration of the freezing events were assessed, analyzing the gait of the patient directly or through a video recorded, but the reliability results of clinical assessment for these features were moderate (Morris et al., 2012; Tripoliti et al., 2013). Percent time frozen seemed to be a reliable metric of severity for both clinical and objective measures (Morris et al., 2012). In addition, the measurement of entropy allowed a detection of FOG events. Furthermore, this method is independent from the type of movement of the patient and the condition of the experiment; it is not based on thresholds and permits the detection of FOG events within longer periods of time while the patient performs daily activities (Tripoliti et al., 2013). Alternatively, the implementation of wavelet transform can have the advantage of providing localization in time and spectral domains, which is important for localizing the FOG events. Furthermore, because the use of a video recording to assess FOG is not always possible, different solutions should be found. The study of Morris et al. (2013) that employed computer-generated animation reconstructed by IMU data could be a promising solution, allowing for monitoring outside of the clinical environment, despite the complexity of this approach and the fact that motion artifacts in the computer-generated representation can affect event detection. Finally, at present, the use of videos seems to be mandatory to obtain good sensitivity and specificity, by establishing a FI (Moore et al., 2008) and thresholds to detect FOG events, because the use of IMU sensors only seems to identify several false positive detections (Zach et al., 2015) that should be avoided. The system for FOG detection should be user-specific (Capecci et al., 2016) and in real time, implementing algorithms able to effectively reduce the delay tolerance between FOG event detection and system reaction to promote a timely intervention (Bächlin et al., 2009) that could help the PwPD to avoid FOG episodes. Moreover, feedback should be context-aware, because continuous cueing is not appreciated by patients (Bächlin et al., 2010) and the efficacy of cueing could decrease over time. An interesting challenge would be freezing prediction instead of freezing detection (Mazilu et al., 2015), whereas possible integration of IMUs with other sensors to measure physiological parameters could provide a more complete analysis of patients' status related to the detection and prevention of FOG episodes, even if current results have limited accuracy (Handojoseno et al., 2012, 2013, 2014, 2015; Mazilu et al., 2015). Finally, the system should be usable outdoors, during unconstrained and unscripted activities (Cole et al., 2011), and be highly compact (Lorenzi et al., 2015), unobtrusive, light weight, easy to use, and meet the requirements of acceptability (Tripoliti et al., 2013; Capecci et al., 2016). In this direction, a smartphone-based system (Capecci et al., 2016) could be a valid solution that could allow patients to use the system during everyday activities and in the community, without discomfort. Also, the solution proposed by Mazilu et al. (2016), which looks for wrist sensors that can be included easily in a smartwatch or wristband, could represent a valid solution in terms of on-body acceptance and accessible technology, despite the fact that it comes at the cost of an increased number of false positives and a slight increase in detection latency.

#### Postural instability

Postural instability is one of the four cardinal motor symptoms in PD, resulting in 14 papers in this review. The pull test is the main clinical examination to assess postural instability, as suggested by MDS-UPDRS, even if the equilibrium score derived from the Sensory Organization Test (SOT) is largely used. Postural instability accounts for 70% of PwPD that fall at least once each year, resulting in an increase of hospitalization and decrease in QoL (Ozinga et al., 2017). Postural control problems cause impairments in PD patients from the early stages of the disease (Gago et al., 2015; Masu et al., 2016). Furthermore, in pharmacologically untreated subjects (Mancini et al., 2011), prediction of postural instability enables prediction of future problems (Palmerini et al., 2011) and slows disease progression (Hubble et al., 2016) (Table 5). Sensor-based systems can improve PD diagnosis both in early disease, by measuring parameters not accurately identified by traditional tests (Hubble et al., 2016), as well as by distinguishing between mild and moderate disease stages (Masu et al., 2016). Pasluosta et al. (2015) proposed a new methodology to estimate postural sway to obtain unbiased and automated score prediction comparable to that rated by the physician. Gago et al. (2015), instead, tried to characterize the postural stability response to normal stance, Romberg test, and Ldopa treatment. PD patients showed high AP postural sway, increased jerk, and low responsiveness to Ldopa, correlated to their gait disturbance, resulting in the ability to identify PD in subjects at the early stage. Contrarily, jerk was slightly close to statistical significance in Hill et al. (2016). In a recent work, Baston et al. (2016) proposed the use of IMUs to quantify postural strategy and sway dispersion among HC and PwPD at different disease stages. Postural strategy was not affected by disease stage, but it was significantly lower in ON compared to OFF medication, and it was associated with self-perception of balance, while sway dispersion was significantly larger in the more severe PD group compared to the mild. In contrast, Mancini et al. (2016) focused on the APA prior to gait initiation and the first step, a state that is not readly observable to the naked eye. The system resulted in high test-retest reliability, both for HC and patients, and data measured from IMUs were highly correlated with those derived by validated systems. The peak of the ML acceleration during APA resulted in the most sensitive measure to the disease, with an amplitude significantly smaller in PD OFF compared to controls. Alternatively, Mellone et al. (2011) determined that since the tremor which is very common in PwPD can affect the identification of postural sway, appropriate techniques of filtering had to be adopted to remove tremor and preserve local dynamics without sacrificing frequency bandwidth. Additionally, the instrumented balance test can be adopted to classify PwPD on the basis of motor subtypes (i.e., dominant tremor or PIGD) (Rocchi et al., 2014) with high accuracy regarding clinical scales. Different motor subtypes show differences in biological and pathophysiological aspects, so their identification can be useful in large clinical studies and to promote accurate personalized therapies. Yelshyna et al. (2016) explored the mechanism underlying compensatory postural adjustments (CPA) by implementing a kinematic and time-frequency analysis based on IMU data during a virtual reality scenario to find differences between PwPD ON, PwPD OFF, and HC, to evaluate Ldopa effects. The lower band (LB) reflected the effect of Ldopa, while the higher band (HB) was responsible for the reaction to visual input-changing scenario; PwPD OFF showed abnormal CPA with respect to PwPD ON and HC in both bands. Finally, Hill et al. (2016) were the first to investigate the relationship of vision and visual-cognition with postural control in PwPD compared to HC. Contrast sensitivity, visuo-constructive ability, and visuo-spatial ability were associated with postural control impairments in PD compared to age-matched HC. Visual biofeedback is important to maintain equilibrium, stability, and vertical body orientation, contributing also to a significantly decreased percentage of falls in PwPD in response to clinical pull test (Caudron et al., 2014).

**Table 5:** Papers about postural instability.

**References**	**Tech**.	**Sensors place**	**Rec. freq**	**Experimental design**	**Subjects**	**Feature extracted**	**Analysis/classifiers**	**Classifier performance or findings**
Ozinga et al., 2017	ACC, GYRO, Neuro Com platform	Waist (COM)	100 Hz	SOT (20 s, 3 trials): SOT1—Fixed surface, EO; SOT2—Fixed surface, EC; SOT3—Fixed surface, sway referenced vision; SOT4—Sway referenced surface, EO; SOT5—Sway referenced surface, EC; and SOT6—Sway referenced surface and vision	14 PwPD, 14 HC	Both for ML and AP directions: peak-to-peak (P2P); normalized path length (NPL); the natural log of RMS distance (logRMS); ellipsoid area (95% Ellipse Area)	ICC and 95% CI; 2-way mixed effects analysis of variance; 2 sample t-Test	ML: ICC = 0.73 for P2P, ICC = 0.81 for logRMS, ICC = 0.87 for NPL. AP: ICC = 0.74 for P2P, ICC = 0.81 for logRMS, ICC = 0.85 for NPL. ICC = 0.863 for ML/AP for 95% Ellipse Area. Postural instability for PwPD greater than HC during SOT5 and with both mobile device (*p* < 0.02) and NeuroCom systems (*p* < 0.05)
Gago et al., 2015	ACC, GYRO	Thighs, shanks trunk	113 Hz	(i) Normal comfortable standing (NS); (ii) Romberg test with EO (REO); (iii) Romberg test with EC (REC). (twice: OFF and ON states, 30 s each)	10 idiopathic, 5 vascular PwPD	Total length of sway, maximal and mean distance of sway with respect to the origin, maximal linear velocity, range of ML and AP sway	Mann–Whitney test; Wilcoxon matched pair test; Spearman test	After Ldopa challenge, both groups differed on MDS-UPDRS III (*p* = 0.001) and mPIGD score (*p* = 0.002). Differences in NS between ON/OFF phases for total length of sway (*p* = 0.027), max distance of sway (*p* = 0.01), range ML sway (*p* = 0.004), range AP sway (*p* = 0.049).
Palmerini et al., 2011	ACC	Lower back	100 Hz	To stand upright, barefoot, with arms crossed on the chest, looking at a visual marker on a wall 2.5 m ahead. EO, EC, EO with dual cognitive task (EODT), EO on a foam-rubber support (EOF), and EC on a foam-rubber support (ECF). (30 s each)	20 PwPD (early-mild), 20 HC	ML and AP directions: power and peak of the PSD in high freq (HF), power ratio of high and low freq (HF/LF); 50% power freq, F95%, centroidal freq, FD, entropy, jerk, normalized jerk, mean and RMS distance from COM trajectory, range of COM displacement, sway path, mean velocity of the COM, sway velocity, ellipse area, angular deviation from AP sway	LDA, QDA, MC, logistic regression (LR), kNN, and SVM with leave-one-out cross validation; Kendall's tau correlation	Range (AP, ECF), Power HF (ML, EODT) and FD (AP, EOF) are the overall highest selection times. Misclassification rate < 5%. *R* = 0.51, *p* = 0.003 between Power HF (ML, EODT) and UPDRS tremor score. *R* = 0.54, *p* = 0.001 between HF/LF (ML, EODT) and UPDRS tremor score. *R* = 0.33, *p* = 0.047 between the deviation from AP sway and UPDRS motor total score
Hubble et al., 2016	ACC	Head, trunk	1,500 Hz	(i) 5 barefoot trials of the TUG test; (ii) 6 barefoot walking trials at a comfortable pace along a 10-m firm walkway (6MTW); (iii) retropulsion falls; (iv) retrospective falls	13 mild (HY = 1) 16 moderate (HY = 2/3) PwPD	AP, ML, and VT HRs	ANOVA or Mann-Whitney test; Spearman's Rho test	More severe motor symptoms (*p* = 0.004), poorer balance confidence (*p* < 0.001), poorer QoL (*p* = 0.001), greater incidence of FOG (*p* = 0.040) and increased PIGD (*p* = 0.002) in moderate PwPD compared with mild PwPD. HRs had significant correlation with retrospective falls for moderate PwPD, while AP HRs of trunk for mild PwPD. ML HRs of head correlated to 6MTW for mild PwPD. HRs of head correlated to the gait and falls questionnaire for moderate PwPD
Hill et al., 2016	ACC	Lower back	Not reported	2-min quiet static stance (looking straight ahead) with feet a comfortable distance apart and hands by the sides	12 PwPD, 10 HC	For ML and AP: sway dispersion, freq of sway, F95%, JERK. For ML/AP: Ellipsis	Spearman correlation	Contrast sensitivity, visuo-spatial ability and postural control impaired in PwPD (*p* = 0.017; *p* = 0.001; *p* = 0.017 respectively). For PwPD only, correlations between higher visuo-spatial function and larger ellipsis (*r* = 0.64; *p* = 0.024) and between impaired attention and reduced visuo-spatial function (*r* = −0.62; *p* = 0.028)
Baston et al., 2016	ACC, GYRO	Shank, lower back	50 Hz	3 repetitions of quiet standing (30 s each) with the arms at the sides looking straight ahead	33 PwPD (HY ≤ 2), 37 PwPD (HY ≥ 3), 21 HC	Postural strategy index (SI), postural sway dispersion as the RMS of the trunk ACC signal in the AP direction (RMS AP)	2-way repeated measures ANOVA; *t*-Tests with Bonferroni correction; Spearman correlation	SI associated with the UPDRS III in PwPD ON (*r* = 0.17, *p* = 0.04), with more hip strategy in PwPD. RMS AP associated with UPDRS III (*r* = 0.25, *p* = 0.03), PIGD subscore (*r* = 0.27, *p* = 0.02) and pull test (*r* = 0.26, *p* = 0.03) in PwPD OFF. SI and RMS AP negatively associated with PwPD OFF (*r* = 0.40, *p* < 0.0001) and ON (*r* = 0.45, *p* < 0.0001). Perception of balance associated to SI in PwPD OFF (*r* = 0.27, *p* = 0.03), and ON (*r* = 0.37, *p* = 0.001). Effect of medication for RMS AP (*p* = 0.002) and for SI (*p* < 0.0001), effect of disease stage for RMS AP (*p* = 0.02). Larger RMS AP in PwPD ON compared to HC (*p* = 0.008)
Mancini et al., 2016	ACC, GYRO, Force plates	Shanks, lower back	128 Hz; 50 Hz	1st study: standing with each foot 10 cm separated. Three gait initiation trials walking at normal comfortable pace, starting with the most affected leg. 2nd study: 3 gait initiation starting with the most affected leg	1st: 10 PwPD, 12 HC. 2nd: 17 PwPD, 17 HC	APA duration, peak ML, peak AP, 1st step length and ROM, 1st step velocity, 1st step duration	ICC; Pearson product moment correlation; *t-*Tests	Differences (*p* < 0.03) in peaks ML and AP between PwPD/HC both for IMU and force plates. 0.42 < ICC < 0.82 with IMU. Good correlation to gold standard measures with force plates and camera (*r* > 0.76). Peak ML is the best sensitive and most reliable measure
Mellone et al., 2011	ACC	Lower back	100 Hz	Standing quietly, barefoot, arms crossed on the chest, and 2.5 m from a visual marker placed on a wall during single-task (ST) and dual-task (DuT)	20 PwPD (early-mild), 20 HC	RMS of ACC, range of ACC, mean velocity, Freq95%, centroidal freq, jerk index (ML direction only)	KW, multiple comparisons *z*-value test with Bonferroni; Linear low pass filter (LPF) and Hilbert-Huang transformation (HHT)	Significant differences: in ST between tremor and non-tremor PwPD for range and centroidal freq for LPF and between all groups for Freq95% and jerk for LPF; in DuT between tremor and non-tremor PwPD for mean velocity both for LPF and HHT, between all groups for RMS, range, jerk index both for LPF and HHT, between all groups for centroidal freq for LPF
Rocchi et al., 2014	ACC	Lower back	100 Hz	(i) Feet together with EO (FT-EO); (ii) feet together with EC (FT-EC); (iii) feet in semi-tandem stance with EO (ST-EO); (iv) feet in semi-tandem stance with EC (ST-EC) (30 s each)	40 PwPD with PIGD, 26 PwPD TD; 15 HC	Power HF (Korczyn and Gurevich, 2010; Caslake et al., 2013; de Lau et al., 2014; Szewczyk-Krolikowski et al., 2014) Hz (2D, ML, AP), HF/LF (2D, ML, AP), centroidal freq AP, mean distance of the total trajectory from the AP signal, sway path (2D, ML, AP), mean velocity of body sway (AP, ML)	ANOVA and Tukey Kramer test; ROC 5-fold cross validation	Differences (*p* < 0.001) for: - power HF, HF/LF between PwPD/HC in all conditions; - power HF between TD/HC; - power HF and HF/LF between TD/PIGD
Yelshyna et al., 2016	ACC, GYRO, Headset	Trunk (COM)	118 Hz	Still standing, barefoot, with the medial aspects of the feet touching each other, arms hanging at their sides and using a safety ceiling trunk belt. Changes in visual virtual reality by using a virtual reality headset device	15 PwPD, 19 HC	SD velocity and max velocity (total, x-axis, y-axis), sway path, sway area, elliptic area, SD ACC, range ACC and average ACC magnitude (AAM) (x,y,z-axes). Time-freq domain: instantaneous mean freq, LB power, HB power	Mann–Whitney test; Wilcoxon matched pair test	Significant differences: - SD ACC, range ACC and AAM on z-axis between PwPD OFF/HC; - SD ACC, range ACC and AAM on z-axis, max velocity and max velocity on y-axis for PwPD ON/HC; - Visual perturbation significantly increased HB power in all groups; - Ldopa significantly increased the basal power
Caudron et al., 2014	Motion Pod^TM^ ACC	Cranial, thoracic vertebras	Not reported	Pull test with EO, EC, and visual biofeedback (BF). Disturbance with verbal instructions: Stability Instruction (SI) and Orientation Instruction (OI)	17 PwPD	Head and AP trunk orientations	*t*-test, ANOVA; Greenhouse-Geisser procedure; Newman—Keuls method	Significant effect for visual condition factor. Final orientation bias lower in BF condition than both EC (*p* < 0.05) and EO (*p* < 0.01). % of fall smaller in BF condition than EC (*p* < 0.05) and EO (*p* < 0.01).

##### Recommendations and trends

Even if the retropulsion test is included in the MDS-UPDRS 3.12 item to evaluate the postural stability, only two studies included in this review attempted to replace this test (Caudron et al., 2014; Hubble et al., 2016), probably due to methodological difficulties. The accelerometer-based approach makes it easier to quantify postural impairments compared to the conventional protocol with force plates, which are more expensive and not portable (Palmerini et al., 2011; Mancini et al., 2016; Ozinga et al., 2017). APA and postural sway, both in ML and AP directions, are the most analyzed features, able to differentiate between PD patients and HC (Ozinga et al., 2017) in the early stages of the disease (e.g., APA disruption can precede the compromission of the step execution) (Gago et al., 2015; Mancini et al., 2016), as well as between mild and severe PD groups (Baston et al., 2016) for differential diagnosis or between ON/OFF states for the advanced patients (Baston et al., 2016; Yelshyna et al., 2016). Alternatively, only one work took into account atypical parkinsonism (i.e., vascular PwPD) other than idiopathic PD patients (Gago et al., 2015), analyzing the response to L-dopa therapy. Additional sensory and attentional demands included in the experimental protocol can be helpful to identify the optimal features for disclosing postural differences between PD and HC subjects (Palmerini et al., 2011).

#### Upper limbs

According to literature, several groups have studied the use of wearable sensors for the analysis of upper limb motion (15 papers were included). In particular, two main approaches have been followed: the use of simple sensors (e.g., on fingers or wrists) or the integration of the sensors in a sort of glove (e.g., TG® medical glove and MiMed smart glove) (Table 6). An alternative solution was proposed by Cavallo et al. (2013) who developed a wireless wearable modular device called SensHand equipped with IMUs placed on the wrist and on the distal phalanx of thumb, index, and middle fingers. The device was not a traditional glove in terms of wearability and modularity, but an integration of inertial sensors. The use of a combination of inertial sensors adequately placed on arms, forearms, hands, and finger segments allowed the measurement of a wide range of parameters both in spatiotemporal and frequency domains. EMG signals, only, were analyzed by Robles-Garcia et al. to assess the variability of the movements in finger tapping (Robles-García et al., 2013). In most of the works, the examiners asked to the patients to perform standard task items described in MDS-UPDRS III (e.g., alternating hand movements, finger-to-nose, finger tapping) for upper limb motion analysis (Fukawa et al., 2007; Okuno et al., 2007; Patel et al., 2009; Yokoe et al., 2009; Hoffman and McNames, 2011; Cavallo et al., 2013; Robles-García et al., 2013; Jia et al., 2014; Delrobaei et al., 2016; Djurić-Jovičić et al., 2016; Eskofier et al., 2016). The papers looked for the correlation between the features extracted and the clinical scores assigned by clinicians on the UPDRS (Okuno et al., 2007) using Pearson (Robles-García et al., 2013) or Spearman correlation (Delrobaei et al., 2016; Djurić-Jovičić et al., 2016), multiple linear regression (Cavallo et al., 2013), ANN (Fukawa et al., 2007), quadratic, and nearest mean scaled classifiers (Djurić-Jovičić et al., 2014a), and they presented a good data separation in clusters between different groups of subjects (Cavallo et al., 2013; Djurić-Jovičić et al., 2014a; Delrobaei et al., 2016). For example, Cavallo et al. (2013) showed better performance in terms of frequency, velocity, and amplitude of movement for HC compared to PwPD, whereas Patel et al. (2009) provided analysis of bradykinesia and dyskinesia with low estimation error values. Also, Eskofier et al. (2016) performed an assessment of bradykinesia, but they introduced the use of deep learning instead of machine learning techniques as a promising method to analyse wearable sensor data. Bradykinesia also was objectively assessed by Delrobaei et al. (2016), who provided for a new index (i.e., BKI score) to express a quantification of this symptoms in upper limbs, according to UPDRS. Djurić-Jovičić et al. (2014a) defined areas and volumes related to tapping activities that can be used to quantify the movement vigor and highlight their decrement over time, which is typical for PwPD caused also by fatigue (Djurić-Jovičić et al., 2016). Yokoe et al. (2009) proposed the opening velocity in the finger-tapping task as a novel parameter for the discrimination of PD patients, whereas Okuno et al. (2007) focused on contact force in finger tapping to predict the level of the pathology. A different approach was proposed by Barth et al. (2012), who used a Biometric Smart Pen (BiSP) equipped with a triaxial accelerometer, finger grip force during holding the pen, refill force, and vibration sound, able to measure handwriting, drawing, and gesture movements on paper or in free air.

**Table 6:** Papers about upper limbs motion analysis.

**References**	**Tech**.	**Sensors place**	**Rec. freq**	**Experimental design**	**Subjects**	**Feature extracted**	**Analysis/classifiers**	**Classifier performance or findings**
Rissanen et al., 2015	ACC, EMG, DBS system	Wrists, shanks	Not reported	For BB muscle: to hold the elbows at a 90° angle with the palms up (20 s). For TA muscle: to keep the toes up keeping the heel on the floor (10 s)	13 advanced PwPD STN-DBS	Sample kurtosis of EMG, recurrence rate (%REC) of EMG, correlation dimension of EMG, coherence parameter between EMG and ACC (only for arms)	*t*-Test; Spearman test	Within-subject differences (*p* < 0.05) for all parameters; *r* = 0.241 *p* = 0.002 for kurtosis; r = 0.336, *p* < 0.001) for correlation dimension; *r* = 0.304, *p* < 0.001 for %REC for correlation to UPDRS arm tremor. *r* = 0.202, *p* = 0.011 for kurtosis; *r* = 0.334, *p* < 0.001) for coherence parameter for correlation to UPDRS arm rigidity
Robles-García et al., 2013	SX230 electrodes, Biometric (EMG)	Extensor digitorum	1,000 Hz	3 blocks of FT with dominant hand (comfort, and slow-comfort rates)	10 PwPD, 10 young and 10 elderly HC	Tapping cycle freq; contact-time; movement-time; CV%; EMG power (RMS) for each burst	ANOVA; Pearson Coefficients	At slow rate imitation lead to a reduction in the variability of movement and induced greater EMG power (*p =* 0.007) in both PD and elderly. Participants reduced their tapping rate at slow comfort vs. comfort self-paced tapping rates (*p* < 0.001). Variability significantly larger in PD than HC. Slow comfort rate CV significantly reduced during imitation in all groups. PD tapped faster than the other two groups (*p* = 0.008)
Delrobaei et al., 2016	ACC, GYRO	Wrists, forearms, arms, shoulders	60 Hz	Pronosupination (10 s)	13 PwPD, 10 HC	Joint angles, SD, angular velocity, time variability, amplitude variability, BKI	ICC; Spearman coefficient; *t*-Test	*r* = 0.626 *p* = 0.001 for BKI/UPDRS correlation. Differences: in BKI between PwPD/HC (*p* = 0.018); in clinical score between PwPD/HC (*p* < 0.001); in BKI between bradykinetic/non-bradykinetic PwPD (*p* = 0.004)
Djurić-Jovičić et al., 2016	ACC, GYRO	Not specified	Not reported	FT (15 s)	13 PwPD, 14 HC, 29 other diseases	Mean, CV and slope of angle amplitude, cycle duration, speed, opening speed, closing speed	ANOVA, Sperman coefficient	Significant (*p* = 0.001) amplitude decrement measured in amplitude slope between PD/HC
Yokoe et al., 2009	ACC, 2 touch sensors	Index finger, thumb	0.1 ms	FT (60 s)	16 PwPD, 32 HC	Mean and SD of: opening and closing velocity, FT amplitude, total distance of FT, freq, FT interval, finger movement interval, finger contact interval	PCA, logistic regression analysis	Mean of opening velocity and FT total distance decreased, while SD of FT interval increased in accordance with deterioration of the UPDRS FT score. Results reported only in box-plots
Patel et al., 2009	ACC (uniaxial)	Forearms, arms, thighs, shanks	100 Hz	Finger-to-nose, FT, hands opening/closing, pronosupination, heel tapping, quiet sitting (30 s, 7 trials)	12 PwPD (HY 2/3)	Range of amplitude (each channel), RMS value of each ACC signal, peak of the normalized cross-correlation function, time lag to such peak value, dominant freq, freq. ratio, signal entropy	SVM (exponential, rbf, polynomial kernels)	SVM with polynomial kernel had the best performance; 5 s window length optimal to achieve minimum estimation error; Estimation error values of 3.4% for tremor, 2.2% for bradykinesia, 3.2% for dyskinesia (dysk)

##### Recommendations and trends

The clear trend in terms of wearable technology is the development of wireless, unobtrusive, quiet, and washable devices that are easy to use without a technician's support (Cavallo et al., 2013). Triaxial inertial sensors seem to be preferable rather than uniaxial accelerometers (Patel et al., 2009) because the former provides the possibility to analyse the motion not only in a plane (e.g., AP plane, sagittal plane) but in a complete 3D space. In addition, combining results obtained by sensor pairs to characterize motion patterns that correspond to normal activity and detect their transition into abnormal ones is easier using triaxial inertial sensors. Further, to acquire data able to finely measure the motion of the hand and fingers, a high sampling frequency of the sensors is needed (e.g., 100 Hz) (Patel et al., 2009; Hoffman and McNames, 2011; Cavallo et al., 2013), instead of low acquisition rate (Jia et al., 2014). Features that are extracted with Continuous Wavelet Transform (CWT) (Djurić-Jovičić et al., 2014a) or entropy (Patel et al., 2009), even if they can discriminate well between PwPD and HC, can be difficult to interpret for clinical staff. Biomechanical measures such as velocity, frequency, or displacement of movements (Yokoe et al., 2009; Cavallo et al., 2013; Delrobaei et al., 2016) provide results that are more appropriate, easier to understand, and more similar to the assessment required by MDS-UPDRS. Halts and hesitations are important parameters, as well, to evaluate the severity of the diseases for tasks such as finger tapping, but no study has proposed their exact calculation based on inertial signals. The calculation of indexes able to quantify the severity of PD symptoms, such as the BKI score for bradykinesia assessment (Delrobaei et al., 2016), are encouraging, because the development of such indicators could overcome the issues of subjectivity and inter-rater variability that currently afflict the diagnosis of the pathology. Furthermore, the implementation of these indexes could assist with home monitoring and personalized assessment and treatment of the symptoms. Further, some features can have a potential use to achieve optimal stimulator settings for DBS, a technique widely used in PwPD, especially in advanced stages of the disease to improve and slow the symptoms of the pathology. Even if DBS effects result in high inter-subject variability, and different DBS settings show high intra-subject variability, the EMG features proposed by Rissanen et al. (2015) could detect motor symptoms that kinematic measurements or clinical evaluation cannot detect, and they can help the clinicians in arriving at optimal DBS settings more quantitatively. To obtain predictive values from motion analysis, Hoffman and McNames (Hoffman and McNames, 2011) proposed a comparison between different adaptive filtering algorithms: least mean square and Kalman filter show the best results in predicting angular velocities and angular values of the movements performed by the patients.

#### Other symptoms: leg agility, rigidity, and arms swing

A task specifically requested by the UDPRS scale for motor evaluation of lower limbs is the leg agility task (LA). It consists of raising the foot from the floor as fast as possible, starting from a sitting posture, for 10 repetitions. A wide number of features can be measured by placing an inertial sensor on each thigh and analyzing this exercise (Giuberti et al., 2014; Parisi et al., 2015), because thigh inclination and heel elevation are highly correlated, as demonstrated by comparing results from the sensors with those of an optoelectronic system. A good correlation emerged between the extracted features and the score assigned by expert neurologists on the UPDRS scale. The use of a wireless body sensor network (BSN) makes the proposed system directly integrable into IoT systems.

Rigidity is one of the four cardinal symptoms in PD, but uncertainty exists about the best method to evaluate it. Rissanen et al. (2009) proposed to measure the dynamic muscle contraction and distinguish between PD patients and HC by analyzing EMG and acceleration signals acquired during elbow flexion and extension movements. The results showed that these dynamic measurements can be informative for assessing neuromuscular dysfunction in PD, even if the accuracy in assessing the patients and the controls was not very high, especially considering that Parkinsonian subjects had low scores in the UPDRS scale for rigidity, finger tapping, and tremor tasks (Table 7). According to the MDS-UPDRS item 3.3, the rigidity should be assessed during passive movements, so methodological difficulties can occur in measuring the rigidity response of PD patients using wearable sensors. This could be responsible for the very limited number of papers found about the measurement of this impairment.

**Table 7:** Papers about rigidity, arms swing and leg agility analysis.

**References**	**Symptom**	**Tech**	**Sensors place**	**Rec. freq**	**Experimental design**	**Subjects**	**Feature extracted**	**Analysis / classifiers**	**Classifier performance or findings**
Rissanen et al., 2009	RIGIDITY	ACC, EMG	Wrists	1,000 Hz	To flex and extend both the elbows vertically and freely in 2-s cycles with the palms up (1 s flexion and 1 s extension)	49 PwPD, 59 HC	%REC, sample entropy, power of ACC, max power of all wavelet coefficients	*t*-Test, KW; Spearman correlation	Correlation (*p* < 0.01, R = −0.443) between the first PCs and total UPDRS motor scores in flexion. 73% sens., 82% spec. for flexion, 80% sens., 87% spec. for extension movements

The assessment of the arm swing using ultrasound-based motion analysis during treadmill walking is useful to identify PwPD, particularly when they are subjected to dual tasks, as well as in response to adaptation of the pharmacological treatment. Specifically, asymmetry indexes based on angular amplitude of the movement calculated for both of the arms were analyzed and compared by Sant'Anna et al. (2011), resulting in good discrimination between PwPD and HC in the first stage of the disease.

### Application 4: Motor fluctuations and on/off phases

Levodopa (Ldopa) is the most common pharmacological therapy adopted by PwPD. It is a treatment able to partially reactivate the neural connections that control the temporal patterns responsible for performing the activity, because Ldopa is converted to dopamine, which is lacking with the progression of the pathology. Also, several side-effects are caused by Ldopa therapy, particularly dyskinesias (referred to as Ldopa-induced dyskinesias or LID), and discrimination between these movements and voluntary motion is difficult to achieve using wearable sensors. PD patients in mid-stage and advanced disease often suffer from motor-fluctuations which represent a severe motor disorder that negatively influences health-related QoL in those patients. Several papers exist in literature about the use of devices based on inertial sensors to monitor the motor fluctuations that affect PwPD, especially in the late stage of the pathology and to evaluate therapy response (12 papers were included in the review). Accelerometers and gyroscope MEMS were placed on different body segments (e.g., wrist, upper arm, thigh, shin, foot, trunk) and combined with other sensors (e.g., EMG or ECG) as reported in Table 8. To monitor the fluctuations that can appear during the day in a patient, both linear and nonlinear features were analyzed. Patel et al. (2009) proposed for monitoring of motor fluctuations; dyskinesia and bradykinesia assessment; and measurements of the intensity of acceleration, modulation, rate, regularity, coordination between right and left sides, and entropy in a continuous monitoring. Cancela et al. (2011), instead, focused on spectral analysis, showing that the power spectrum in PwPD is wider than in HC, and the power in the main peak moved to different frequency bands, generating new peaks with a significant power. Samà et al. (2012) also focused on the spectral analysis of the accelerometer signal, defining frequency thresholds able to identify if a patient suffers from dyskinesia, avoiding false positive detections, similarly studied by Pérez-López et al. (2016). Further, they provide for an ON/OFF state algorithm detection, based on stride characterization during walking, since OFF states results in lower amplitude in both temporal and frequency domain. To evaluate the pharmacological therapy taken by the patients, instead, Ruonala et al. measured ECG-derived parameters both in time and frequency domain, demonstrating that some parameters effectively decrease in a significant way, between off and on medication (Ruonala et al., 2015). Hssayeni et al. (2016) focused on signal power, jerk, entropy, peak-to-peak, and correlation coefficient and developed a semi-supervised clustering approach, k-means based, to automatically assess the ON/OFF medication states of PwPD. Finally, Ramsperger et al. (2016) measured dyskinesia as the ratio of the angular rate around the z-axis over the angular rates lying within the xy-plane, as measured by the triaxial gyroscope sensor within the SENSE-PARK European research project. The previous features were extracted following specific experimental protocols that included standard diagnostic exercises according to the UPDRS scale such as finger-to-nose, tapping, sit-to-stand, walking, stand-to-sit, finger tapping, alternating hands movements, heel tapping (Patel et al., 2009; Rissanen et al., 2011); or allowing subjects to do everyday free activities (e.g., walking, reading, eating) (Ramsperger et al., 2016); or prescribing specific daily activities (e.g., cutting food, dressing) Samà et al. (2012) (Hssayeni et al., 2016); or permitting a combination of both typos of movements (Pastorino et al., 2011; Rahimi et al., 2011; Tsipouras et al., 2012) eventually with some restrictions (e.g., subject seated) (Tsipouras et al., 2011). The aim of these studies was to identify different motor states (Patel et al., 2009; Samà et al., 2012; Hssayeni et al., 2016); quantify the efficacy of treatment (Ruonala et al., 2015) and DBS (Rissanen et al., 2011) in PD to assess the severity of bradykinesia (Pastorino et al., 2011), dyskinesias (Pérez-López et al., 2016; Ramsperger et al., 2016), and LID (Tsipouras et al., 2011); and to manage them (Tsipouras et al., 2012). For this reason, permitting the use of the system at home in an unsupervised environment, at times, is important (Pastorino et al., 2011; Ramsperger et al., 2016).

**Table 8:** Papers about motor fluctuations analysis.

**References**	**Tech**.	**Sensor place**	**Rec. freq**	**Experimental design**	**Subjects**	**Feature extracted**	**Analysis/classifiers**	**Classifier performance or findings**
Pérez-López et al., 2016	ACC	Waist	200 Hz	1st study: activities guided but execution free (e.g., indoor/outdoor walking, FOG provocation, dysk, false positive tests), (before/after medical intake). 2nd study: Lab activities (walking in a straight line, over an inclined plane, carrying a heavy object, setting a table, going upstairs and downstairs) and outside protocol (walking for >15 min)	1st: 92 PwPD. 2nd: 10 PwPD (mild to moderate and motor fluctuations)	Power spectrum in dyskinetic band (0.68–4 Hz), non-dyskinetic band (8–20 Hz) and posture transitions (0–0.68 Hz)	SVM (leave-one-out-subject)	100% sens., 98% spec. for strong trunk dysk; 39% sens., 95% spec. for weak dysk on limbs; 93% sens., 95% spec. for all strong dysks or weak dysk on trunk
Ramsperger et al., 2016	ACC, GYRO	Ankles	Not reported	Everyday activities: making coffee, lying, sitting quietly, sitting and counting coins, dressing. 1st study: 6 h, lab-environment; 2nd study: 12 weeks in home-environment	1st: 23 PwPD (7 leg dysk), 13 HC; 2nd: 10 PwPD (7 dysk)	Ratio of the angular rate around the z-axis over the angular rates lying within the xy-plane	*Ad hoc* algorithm for dysk detection; correlation coefficient	85% sens., 98% spec. 0.96 acc. for dysk detection in lab-environment; Perfect discrimination in home-environment; 0.61 (*p* < 0.001) correlation with UPDRS
Rissanen et al., 2011	ACC	Wrists	Not reported	(i) Isometric task: to hold the elbows at 90°angle with the palms up (30 s); (ii) dynamic task: elbow flexion / extension movements in VT direction within 2-s cycles with the palms up	13 PwPD, 13 HC	Correlation dimension of EMG, %REC of EMG, RMS amplitude of ACC, sample entropy of ACC, coherence between EMG and ACC	PCA	EMG of patients changed into more complex and contained less recurring structures due to DBS. Amplitude and regularity of ACC, coherence between EMG and ACC and side differences (left/right) reduced due to DBS
Tsipouras et al., 2012	ACC, GYRO	Wrists, legs, waist, chest	62.5 Hz	Lying, rising from the bed and sitting on a chair, standing up and perform (8 min): walking, opening/closing a door, opening the door and walking in the corridor, returning to the room, making a stop and drinking from a glass, returning to the chair and sitting	11 PwPD (6 LID), 5 HC	Mean, SD, mean entropy, signal energy in different bands, spectral entropy, spectral SD from each axis of each sensor	DT C4.5	80.35% acc., 76.84% Positive predict value for LID severity

#### Recommendations and trends

The study and identification of motor fluctuations in PwPD is a challenge in the long-term management of the pathology. The OFF states can appear during the day, when the effect of the drugs consumed by patients is reduced and the severity of the symptoms comes out or re-emerges. When a patient is in the OFF phase, the patient's condition can be considered critical, and the patient can be subject to the risk of falls, FOG events, LID, significant tremor, and general difficulties in performing daily activities. Commonly, this situation is not manifested when a patient is under medical examination in a hospital or in ambulatory monitoring, so the ability to control the subject at home throughout the day is essential to identify and to prevent, if possible, these critical events. The pharmacological treatment most commonly used for PD is based on Ldopa. This drug effectively holds off the motor symptoms of the pathology (Ruonala et al., 2015), but at the same time, it can be responsible for side effects such as LID that, in turn, can be very disabling for PwPD and predictive for risk of falls (Ramsperger et al., 2016). An adequate monitoring of LID is needed to adjust the pharmacological treatment followed by the patients and ensure the benefits derived from an optimal drug therapy (Pérez-López et al., 2016). However, the detection of LID severity, particularly for slightly impaired patients, is not easy to obtain (Tsipouras et al., 2011). Because of the lack of objective methods for quantifying the efficacy of treatment in PD, new strategies should be implemented (Rissanen et al., 2011; Ramsperger et al., 2016). Since the border between ON and OFF depends on the stage of the disease and on the patient, thresholds that best distinguish both motor states in a certain patient are not expected to be the best thresholds for another patient (Samà et al., 2012). The inter-individual differences between patients, in fact, can lead to different responses both on therapy treatment and motor states. To date, the best results in identifying different motor states are restricted to patients with a great improvement in tremor and bradykinesia from medication OFF to ON stage (Hssayeni et al., 2016). Long-term experimentations in home settings appear necessary to obtain valuable data that allow an accurate assessment of ON/OFF motor states in PwPD and dyskinesias (Ramsperger et al., 2016).

### Application 5: Home and long-term monitoring

To date, as clinical scales are the gold standard for in-clinical setting assessment of PD, the use of patient-completed symptom diaries is the current gold standard for the home monitoring of the pathology (Fisher et al., 2016). Recent studies proposed the use of commercial devices such as the Microsoft Kinect sensor (Paredes et al., 2015) as a low-cost solution to assess the movement of Parkinsonian patients, not only in clinical settings, but also at home. Nevertheless, the accuracy of these systems can be considered good in the measurement of spatiotemporal features for gross movements, but it is not acceptable compared to validated motion capture systems, which are the gold standard for fine movement analysis of actions such as hand clasping or finger tapping, which is required in the MDS-UPDRS scale for PD severity evaluation.

The concept of monitoring patients in their own homes is the future trend in terms of long-term monitoring, instead of the typical ambulatory monitoring proposed in previous works (Salarian et al., 2007a), even if with good results. Recently, the European Committee has emphasized this direction, as demonstrated by the grant of different projects for home monitoring. PERFORM, for example, consisted of design, development, validation, and exploitation of a multi-parametric system for the long-term, continuous, and effective assessment and monitoring of motor status in PD using the PERFORM WMSMU, a wearable multi-sensor monitoring unit (Tsipouras et al., 2014). The system aimed to allow the physicians to monitor and detect changes in the symptomatic behavior as quickly as the changes appear (Cancela et al., 2010). Also, the European research project CuPiD looked for home environment applications. In particular, within this project, a system composed of IMUs and smartphone-based application was developed with the aim to provide an efficient gait training application at home for PwPD (Ginis et al., 2016). They system gave real-time measurement of gait, auditory biofeedback on spatiotemporal gait parameters, and rhythmical auditory cueing to prevent or overcome FOG episodes. The system was able to improve gait and balance in PwPD in a more effective way than traditional home-based gait intervention as well as follow-up controls. Moreover, it appeared well-tolerated and user-friendly for PwPD, even for those who were unfamiliar with a smartphone. The systems proposed in literature (14 papers included in this review) for monitoring and managing the development of the disease at home are equipped with triaxial accelerometers and gyroscopes, MEMS or EMG devices, placed on different body segments (e.g., wrist, ankle, waist, thigh, shin) for a full wearable system that did not impose limitations to patients' movements (Table 9). The data logger was required to be portable and multifunctional, and a robust control on the device was adopted to inform the patient about the treatment program or to allow the sending of emergency calls if needed (Tsipouras et al., 2014). Alternatively, Cook et al. (2015) proposed the CASAS Smart Home, in which sensor data from ambient sensors are added to wearable sensors and smartphone. The system included a large number of sensors, but it allowed extraction of a considerable number of features, revealing differences between tasks performed by HC and PwPD and providing an automatic classification of end users between HC, subjects with mild cognitive impairment (MCI), and PwPD with or without MCI. To control the development of the disease, different approaches are adopted, as reported in literature. In some works the patients are asked to perform standardized motor tasks only, as those described in the motor section of MDS-UPDRS (e.g., quiet sitting, finger tapping, finger-to-nose, alternating hand movements, heel tapping, walking) (Patel et al., 2009; Jaywant et al., 2016). Other works required instead the performance of ADL or similar common everyday tasks (i.e., preparing snacks, eating, reading, writing, using of Internet, conversing with someone) (Cancela et al., 2010; Rahimi et al., 2011; Khan et al., 2014; Lambrecht et al., 2014; Cook et al., 2015; Fisher et al., 2016), whereas further works aimed to continuously monitor the patients (Pastorino et al., 2011; Roland et al., 2013, 2014; Wallén et al., 2014), eventually even during the night (Fisher et al., 2016). In terms of measurements, a wide range of features were extracted for long-term monitoring at home, including statistical and frequency features, gait parameters, Fourier coefficients, and many others.

**Table 9:** Papers about home and long-term monitoring.

**References**	**Tech**.	**Sensors place**	**Rec. freq**	**Experimental design**	**Subjects**	**Feature extracted**	**Analysis/classifiers**	**Classifier performance (findings where classification is N/A)**
Fisher et al., 2016	ACC	Wrists	100 Hz	1st study: 4 h in lab performing MDS-UPDRS III items (4, 6, 10, 11, 15 e 17) for upper limb bradykinesia, tremor and gait. 2nd study: 7 days at home performing ADLs	34 patients	91 features including: Fourier coefficients, empirical cumulative distribution function features and statistical features	ANN leave-one-out. Correlation coefficients	Dysk assessment: 0.38 sens., 0.99 spec. in lab, 0.49 sens., 0.93 spec. at home; ON–OFF detection: 0.65 sens., 0.83 spec. in lab, 0.51 sens., 0.87 spec. at home; Correlations: UPDRS IV/dysk *r* = 0.52, *p* = 0.008 (excellent diaries), *r* = 0.52, *p* = 0.004 (good diaries); UPDRS IV/ON–OFF not significant; diaries/dysk *r* = 0.69, *p* = 0.001 (excellent diaries), *r* = 0.65, *p* = 0.002 (good diaries); diaries/ON–OFF: *r* = 0.63, *p* < 0.004 (excellent diaries), *r* = 0.56, *p* < 0.01 (good diaries)
Ginis et al., 2016	ACC, GYRO, smart phone	Shoes or ankles	100 Hz	Gait training for 30 min, 3 times per week for 6 weeks	20 PwPD, 18 HC	Gait speed, stride length, DS time	*t*-Test. Fisher's LSD *post-hoc* analysis for effect size	*p* < 0.001 for gait speed and stride length, *p* < 0.01 for DS time both for PwPD and HC. PwPD improved more than HC both in comfortable and dual task condition. Effect size: small for HC, moderate for PD
Cook et al., 2015	ACC, GYRO, CASAS Smart home, smart phone	Upper dominant arm, ankle of dominant side	30 Hz	IADLs: water plants, medication management, wash countertop, sweep and dust, cook, wash hands, TUG test, TUG test with name generation, Day Out task	1st: 25 PwPD, 50 HC; 2nd: 16 PDNOMCI, 9 PDMCI, 9 MCI, 18 HC	Ambient sensor features, wearable sensor features, Day out task features, participant features, activity features	DT, Naïve Bayes, RF, SVM, Ada/DT, Ada/RF. ANOVA, ROC	0.74 AUC, 0.75 acc. (individual tasks), 0.70 AUC, 0.70 acc. (combined tasks) for PwPD/ HC recognition with Ada/RF. 0.96 AUC, 0.85 acc. (individual tasks), 0.64 AUC, 0.32 acc. (combined tasks) for PDNOMCI/PDMCI/MCI/HC recognition with Ada/DT. Best performance using all sensors and all activities
Jaywant et al., 2016	ACC	Ankles	100 Hz	Walking trials: straight line walking (10 m/20 m), walking with turns (16 m), walking to sit in a chair (18.8 m/13.8 m)	23 PwPD (walking difficulties)	Walking speed, stride length, stride freq, leg swing time, gait asymmetry	*t*-Test. Cohen test for effect size	Increased post-training self-reported mobility (*p* < 0.05) in gait observation group. Greater improvement in walking speed and stride length (not significant, medium effect) for gait observation group than landscape group
Wallén et al., 2014	ActiGraph GT3X+ ACC	Waist	30 Hz	1st study: step count assessment in controlled setting*:* walking, self-paced speed, 3 min along a 2 × 150 m long hallway. 2nd study: free-living physical activity: 60–90 min session of motor and cognitive testing for a week	1st: 15 PwPD. 2nd: 65 PwPD	# steps, step count, time spent in sedentary behaviors, low/moderate intensity activities, moderate-vigorous intensity ambulatory activities, total wear time	*t-*test; ANOVA, Wicoxon signed-rank. Pearson and Sperman coefficients; ICC, CI	No significant difference in # steps measured by ACC/video. LF Extension (LFE) filter consistently generated larger values than Normal Filter on all outcome variables. LFE might overestimate outcomes in free-living conditions
Roland et al., 2013	EMG Biometrics, ActiTrainer ACC	EMG: BB, triceps brachii (TB), vastus lateralis (VL), biceps femoris (BF). ACC: waist	1,000 Hz	Maximal handgrip and maximal voluntary exertion (MVE) for each muscle group 1–2 h post-anti-PD medication or following breakfast (HC). Adherence to typical daily routine encouraged except for water-based activities (6.5 h). Then maximal handgrip, MVE and remove equipment	23 PwPD, 14 HC	MVE for each muscle group. EMG gap (defined as < 1% amplitude of MVE for >0.1 s as #, duration and time occupied by gaps)	*t*-test; 2-way ANOVA; 3-way mixed measures ANOVA	Gap duration shorter (*p* = 0.04) and occupied less time (*p* = 0.02) in PD than HC and in females compared to males (*p* = 0.004). Gaps fewer (*p* = 0.04) and occupied less time (*p* = 0.01) on more-affected than less-affected side. PD weaker than HC (*p* = 0.04), females weaker than males (*p* = 0.00), and the more-affected PD side weaker than less-affected (*p* = 0.04).
Roland et al., 2014	EMG device Biometrics				13 PwPD	# gap, duration and %; # burst, duration, amplitude and %, MVE for each muscle group	ANOVA	Burst duration and burst % were significantly different between frail and non-frail states. Frail females had 73% decreased gaps and 48% increased burst duration compared with monorail

The system proposed by Fisher et al. (2016) was composed of wrist-worn accelerometers to acquire data in laboratory and home environment and ANN to compare the sensor data with the diaries of patients as self-reported. Diaries and clinician-rated assessments were compared. High specificity both in laboratory and at home was seen for dyskinesia, but with low sensitivity at home, perhaps because the system is based on wrist-worn accelerometers. Thus, dyskinesia that can occur in other body segments cannot be assessed. High correlation was found between sensor data and diaries regarding the amounts of time in a given disease state. However, since the ideal PD home monitoring system should be real time and adaptable, and in light of the information obtained, the real-time evaluation with the proposed sensor system is not feasible. A different analysis was conducted by the research group of Roland et al., who pointed out the differences between PwPD and HC, but also between genders on the basis of features extracted by EMG signals, and found significant dissimilarities (Roland et al., 2013, 2014). Finally, Jaywant et al. (2016) studied the efficacy of home-based gait observation training to enhance walking in PD. Objective changes in gait did not result, but an increased self-perceived mobility was reported. Since this aspect is evaluated through the administration of PDQ-39 mobility subscale, it is possible that accelerometers did not measure the kinds of functional improvements perceived by the patients and expressed in the clinical scale. Furthermore, the system was feasible and easy to understand and to use.

#### Recommendations and trends

The principal aim of the home monitoring is to provide an optimal management of PD. According to literature results, this can be done by observing the development of the pathology through the analysis of data acquired by wearable sensors, which seem to be the best type of devices to adopt. The implementation of a Smart Home, in fact, lowered the users' acceptance of the technology and resulted in an invasive system that did not provide a sufficiently high accuracy in observations and also measured numerous irrelevant features (Cook et al., 2015).

To obtain a useful monitoring system, the clinicians should be able to interact remotely with patients in the home setting, to configure the sensor nodes for the application at hand, and to record annotated data with the possibility of video conferencing, real-time data visualization, data collection supervision, annotation tools, and spot checks. Collecting data in the home setting and remotely monitoring the patient would allow clinicians to improve quality of care of the patients while reducing the costs. From a technical point of view, the system should be able to work in an unsupervised environment (Pastorino et al., 2011; Roland et al., 2013, 2014; Wallén et al., 2014; Fisher et al., 2016), provide real-time biofeedback (Ginis et al., 2016), feature user-friendly interfaces both for clinicians and patients, and attend to the real-time transmission of the data acquired to prevent data loss caused, for example, by absence of available Internet service, to be acceptable and usable (Fisher et al., 2016; Ginis et al., 2016; Jaywant et al., 2016).

At the moment, the large variability resulting within subjects for each task, across tasks for individual subjects, and between scripted and unscripted tasks is a crucial point to overcome to ensure the correct assessment of the status of the patient. The use of a large number of sensors seems, for instance, the most feasible solution to capture the wide range of movement patterns adopted by PwPD to perform required tasks (Rahimi et al., 2011). On the contrary, to address acceptability and usability requirements, a reduced number of sensors is more appropriate, as with the wrist-worn devices proposed by Fisher et al. (2016), which are usable also in the home environment and do not compromise the execution of common ADL. Moreover, large dimension and weight of wearable devices should be avoided because they are not comfortable (Pastorino et al., 2011).

For future works, the system should be totally automatic and avoid the requirement that patients must introduce information manually about medication or meal intake using the developed GUI, or compile a daily diary (e.g., every half-hour). Finally, to obtain real-time assessment of the patients' status, different machine learning methods were, and the SVM with Gaussian radial kernel seemed to be the best classifier for detecting PD (Khan et al., 2014).

## Discussion and conclusions

PD is a disabling pathology that affects millions of people worldwide. Since the disease heavily influences the QoL of patients, raising the burden of care on their relatives and the costs for health and care for the society, an optimal solution for the management and treatment of PD is needed. This paper focuses on the use of wearable devices for PD applications (early diagnosis, tremor, body motion analysis, motor fluctuations and ON–OFF phases, and home and long-term monitoring), to analyse the current state-of-the-art of existing systems used in this field and to identify the pros and cons for each work with the aim to give recommendations for future improvements.

Currently, PD is diagnosed when wide areas of the brain are already damaged, because the patients go to the clinician when motor symptoms are evident and begin to influence their common activities. Thus, the diagnosis is generally made when the brain neurodegenerative process is already triggered, whereas to improve the treatment of the pathology, PD should be diagnosed when it is at the onset. Recent trends show that it is very important to identify the disease in the early stage, possibly in the prodromal phase, when the symptoms are not yet evident, to optimize the management of the pathology and to improve the quality of care and consequently the QoL for the patients. Currently, the neurologist diagnoses the pathology by asking the patient to perform tasks defined in the MDS-UPDRS (Goetz et al., 2008) and assessing the patient through a visual examination. The scale adopted is semi-quantitative, and the neurologist assigns a score between 0 (normal state) and 4 (severe stage of PD) for each task performed and then sums the scores for all the items. This type of evaluation is subjective, based on the experience of the clinician, and generally it can vary between different neurologists and health centers, making the diagnosis inaccurate or uncertain, at times. Additionally, to confirm the diagnosis, invasive and expensive nuclear medicine tomographic imaging techniques are generally adopted (i.e., single proton emission computed tomography—SPECT—with DaTscan) with high costs for healthcare. For these reasons, in recent years, different research groups have worked to find a method to objectively measure the motor performance of the patients, since the motor symptoms are those that generally lead the neurologist to the diagnosis. The idea is to quantify the motor skills of the patients, finding a way to measure the items proposed in MDS-UPDRS III. A method to objectify the motion can lead to a quantitative diagnosis of the PD, overtaking the problems linked to the subjectivity and to the inter-rater and intra-rater variability, thus increasing the accuracy of the diagnosis. A study (Ghassemi et al., 2016) revealed that the overall classification rate is not only limited by technical accuracy, but also by clinical accuracy. In this direction, different works proposed well-defined experimental protocol to replicate the MDS-UPDRS III items, looking for a close correlation between the parameters measured with the technological solution adopted and the clinical scores assigned by the neurologist. System cost and portability are two important characteristics that must be considered when developing a novel diagnostic tool. To measure the motor performance of the patients, the most appropriate way seems the use of wearable devices based on inertial sensors, which can acquire data with a high sample rate (e.g., 100 Hz, Mellone et al., 2011; Palmerini et al., 2011; Cavallo et al., 2013; Sejdić et al., 2016), and examination of the results on board or transmittal of results to a control station for offline data processing. Thanks to the recent advances in MEMS technology, this type of device is portable, light weight, unobtrusive, easy to use, inexpensive, and accurate in the measurements. Thus, wearable devices with inertial sensors can represent an optimal solution in healthcare applications, for use in both clinical infrastructure and the home environment. Similarly, traditional motion capture tools such as multi-camera retro-reflective motion analysis systems, while potentially effective at finely measuring the motor performance of the subjects, are nonetheless difficult to bring out of a laboratory setting because of their cost and non-portability. Although features extracted from 3-D motion data are slightly more accurate than features extracted from inertial sensor data, inertial sensors are non-invasive and can be used continuously and in uncontrolled environments (Sant'Anna et al., 2011).

The possibility to use the system not only in controlled health infrastructures, but also in unsupervised environments (homebound setting, over a 24-h cycle) (Pastorino et al., 2011; Fisher et al., 2016; Ramsperger et al., 2016) and during unscripted tasks (Cole et al., 2011; Roy et al., 2011) is crucial to improve the monitoring and assistance for PD patients during common everyday activities. For this reason, the devices must be tested fully, not only for the typical items proposed in MDS-UPDRS III, which are needed for the diagnosis, but also for the ADL and other tasks of everyday life (Salarian et al., 2007b; Bächlin et al., 2009, 2010; Mazilu and Hardegger, 2012; Lambrecht et al., 2014; Cook et al., 2015; Fisher et al., 2016; Rigas et al., 2016). Since video recording for long-term monitoring during free daily activities is not possible (Salarian et al., 2007b), the use of wearable sensors is the most promising solution for this purpose, with the plan to eventually implement them into IoT systems (Giuberti et al., 2014). An alternative approach in order to avoid high variability in remote monitoring could be the use of Virtual Reality technologies. They, in fact, could be an useful tool for an homogeneous approach of monitoring patients at home and they could be suitable also for other applications (i.e., rehabilitation) in PD (Dockx et al., 2016).

Moreover, to guarantee an optimal management during the home monitoring, it is mandatory to develop and provide user-friendly interfaces that allow the clinicians and the patients to stay in contact (Pierleoni et al., 2014), adopting a sort of telemedicine service that permits exchanging of information, consulting service, and therapy adjustments. At the beginning, patients, caregivers and medical staff should be trained to use this technology, adopting educational strategies that could increase the level of confidence with the proposed solutions. Then, with automatic updates to a mHealth server, caregivers and healthcare professionals can gain insights into overall wellness of the subjects by analyzing the parameters from multiple tests performed in a single day or monitoring and evaluating the evolution of disease by analyzing the trends in the parameters collected over longer periods of time. In this direction, the recent EU PD_Manager Project (Rigas et al., 2016) was targeted the development of a complete mHealth PD management solution through Microsoft band and Android application. The real-time (Ferrari et al., 2016) and automated assessment of the performance of the patients (Giuberti et al., 2015) is another cardinal point for the development of an efficacious remote support and monitoring system. It allows assessment of the status of the patients on demand, evaluating also the eventual changes due to modification in pharmacological or rehabilitative treatment, as well as objective evaluation of the efficacy of the adopted therapy. In fact, it is widely recognized that self-report assessments can be limited by patients over- or under-reporting their difficulties. Hence, more objective tests would greatly benefit the clinical assessment of PwPD (Hubble et al., 2016). Finally, an adequate monitoring and assistance system at home could reduce the number of medical examinations in hospital, promoting the empowering and the self-consciousness of the PwPD in the management of their own disease, and stimulating them to improve the performance in following a personalized therapeutic pathway in developing specific models for home monitoring.

As reported in Table 10, a significant limitation of most studies reported in literature is the small dataset adopted to test the technological solutions proposed (Figure 2D). The small sample size reduces the generalizability of the results, which should be verified in a larger sample (Mariani et al., 2013; Palmerini et al., 2013) and, specifically, longitudinal and large sized validation is needed to prove clinical applicability (Ghassemi et al., 2016) of the developed technologies.

**Table 10:** Limitations and opportunities.

**TYPE**	**Barriers/limitations**	**Challenges and opportunities**
Experimental	Dataset	High number of subjects must be recruited to validate the results of the studies.
	Dataset	Patients with mild impairments (HY = 1) should be investigated to promote early diagnosis.
	Dataset	Age-matched controls should be involved, because motor performance vary with age.
Technological	Acceptability	The system should be developed with consideration for patients' requirements and needs.
	Usability	The system should be easy to use and eventually provide user-friendly interfaces both for clinician and patients.
	Portability and unobtrusiveness	The system should be wireless and non-invasive for use at home or outdoors without limitations for the patients. The number of sensors used and their placement over the body should address the trade-off between accuracy in measurements and obtrusiveness.
	Long-term monitoring	The system should ensure long-term operation. Long-term battery and low-power solutions have to be adopted, especially for home-monitoring applications for advanced patients with motor fluctuations.
	Measurements	The system should provide quantitative, accurate, precise, objective, and reliable measurements of the symptoms analyzed to actively support the clinician in diagnosis and management of the pathology.
		The system should be able to recognize different stages of the disease as well as to evaluate changes due to pharmacological treatments.
Clinical	Availability for use in clinical practice	- Accuracy, precision, reliability, and dependability of the system should be ensured. - Objective support for clinical examination and assessment should be validated, complying with traditional medical scales. - Improvements for patients' management should be efficiently demonstrated.

Another open issue in the analyzed works concerns the optimal number of sensors to use for recording patient activities. The literature has a lack of consensus regarding the optimal number of sensors, and the optimal site for their placement, for the assessment of PD motor symptoms (Fisher et al., 2016). The sensors should guarantee that the subjects can perform the movements without restrictions. If a reduction in the number of sensors may lead to loss of potentially relevant information (Pastorino et al., 2011), to avoid a high invasiveness of the system, a restricted number of sensors must be used (Salarian et al., 2013), because using up to three on-body sensors together with a phone decreases the acceptance of the system (Mazilu et al., 2016). Thus, a trade-off must be found and adopted. The acceptability of the system would be considered, particularly for smart home monitoring and long-term assessment of motor symptoms (Barth et al., 2011), when a prolonged use of the devices is required. In this direction, recent studies (Arora et al., 2014; Kostikis et al., 2015; Capecci et al., 2016; Ferrari et al., 2016) proposed the use of smartphones that are equipped with inertial sensors. This device is a common technological tool that does not require specific technical capabilities for the use and is widely accepted and usable, not only in healthcare infrastructures, but especially at home, outdoors, and in the community.

Moreover, the features to extract from the inertial signals and to provide to the clinical staff are a matter of debate. Generally, they are selected by using statistical techniques such as ANOVA or the Mann–Whitney test, which can identify the features that best discriminate between different subjects' groups (i.e., patients and HC). Even if this is mathematically correct, the features proposed are not always easy to understand by the clinicians. It is important, instead, to provide the clinical staff with a reduced and comprensive set of features to avoid misleading in clinical practice (Palmerini et al., 2011).

Finally, to provide automatic (almost real time) assessment of the performances measured, recent works implemented different machine learning approaches (Kostikis et al., 2015), such as SVM (Patel et al., 2009), NaiveBayes, kNN, NCC, RF (Reinfelder et al., 2015), decision tree (DT), and LDA (Barth et al., 2011; Perumal and Sankar, 2016). All of these classifiers were used to discriminate between PwPD and patients with similar symptoms but different pathologies (e.g., subjects with ET) (Surangsrirat et al., 2016) or to distinguish between PwPD and HC, or to identify different stages of the disease, or to evaluate the medication and DBS effects (Rissanen et al., 2011). In various works, the Principal Component Analysis (PCA) is implemented as well, generally for the dataset reduction and feature selection (Palmerini et al., 2011; Parisi et al., 2015), or for visual inspection analysis. In fact, the reduced-dimensional system of the principal components could be used for explorative analysis (e.g., for clustering subjects with the same behavior) and to detect outlier subjects, which have a performance very different from the average. This technique is often used in combination with the discussed machine learning techniques (Rissanen et al., 2009; Tien et al., 2010; Giuberti et al., 2015; Parisi et al., 2015; Ghassemi et al., 2016).

Alternatively, a recent work proposed the use of deep learning as a promising method to analyse wearable sensor data in place of machine learning approaches (Eskofier et al., 2016). Advantages of deep learning are (i) there is no need to rely on expert-defined features that may or may not represent the information content of the signal that is subjected to classification; (ii) the analysis procedure resembles what human experts do, since the whole signal segment is rated with one continuous and clinical scale-like output; (iii) an adaptation of the network to an individual patient is possible; (iv) deep learning based frameworks, in particular, can be expected to produce better results with the growing amount of data that will become available. However, to manage these data, high quality labels are required from clinical experts. Deep learning may provide higher accuracy than machine learning (e.g., AdaBoost.M1, RF, kNN, SVM) for correctly classifying symptoms and subjects, but further and extended studies are required to validate this theory.

In conclusion, this review provides a wide overview of the technological solutions currently implemented for PD applications. The rising idea from literature is to use unobtrusive and accurate systems that could monitor the disease progression since its beginning, throughout its development. Appropriate technological solutions, in fact, could improve the management of the PD, enhancing the QoL of PwPD, through an early and objective diagnosis of the pathology, as well as monitoring the effects of the pharmacological therapy during the disease progression.

## Author contributions

ER was responsible for paper structure and writing, synthesizing the information from the papers into text and tables. CM was the clinical supervisor, responsible for clinical aspects and contributing in introduction, methodology definition and search strategies. FC was the scientific supervisor, guarantor for the review and contributed in methodology definition, paper writing, discussion and conclusion. All authors were involved in papers screening and selection. All authors read, provided feedback and approved the final manuscript.

### Conflict of interest statement

The authors declare that the research was conducted in the absence of any commercial or financial relationships that could be construed as a potential conflict of interest.
