# Supplementary material for: How Wearable Sensors Can Support Parkinson's Disease Diagnosis and Treatment: A Systematic Review
Source: Front Neurosci. 2017 Oct 6;11:555. doi: 10.3389/fnins.2017.00555 (PMC5635326; doi:10.3389/fnins.2017.00555)
Supplement: Supplementary file 1 [file Table1.pdf]

Table 1b. Conference papers about early diagnosis.

Ref	Tech.	Sensors Place	Rec. Freq	Experimental Design	Subjects	Feature extracted	Analysis / Classifiers	Classifier Performance or Findings
Brodie et al. 2014	ACC	Head, pelvis	128 Hz	19 m walkway	10 PwPD (mild), 10 HC	Jerk, harmonic stability, oscillation range	ANOVA; PCA; Pearson correlation	PwPD presented faster AP head movements (p=0.02) and slower walking speed (p=0.02) than HC

Table 2b. Conference papers about tremor analysis.

Ref	Tech.	Sensors Place	Rec Freq	Experimental Design	Subjects	Feature extracted	Analysis / Classifiers	Classifier Performance or Findings
Ghassemi et al. 2016	ACC, EMG	ACC: hands. EMG: forearms	1000 Hz	Resting task; Postural task; Postural task with 1 kg attached to forearm (each 30 s)	13 PwPD (tremor dominant form), 11 ET	mean, SD, skewness, kurtosis, entropy, energy, RMS, mean absolute value	DWT method PCA, SVM	Acc.: 79% for RT; 75% for PT; 83% for PT with weight
Surangsrirat et al. 2016	ACC, GYRO	Wrist and Forefinger tip	125 Hz	Resting task; Kinetic task (finger-to-nose movement) (each 10 s)	32 PD tremor, 20 ET	Temporal fluctuation of tremor signal	SVM 10-fold cross validation	100% sens., 100% spec.; 100% acc. for PD/ET classification
Niazmand et al. 2011	Smarth clothes	ACC in MiMed pullover	20 Hz	Resting task; Postural task (each 15 s)	10 PwPD, 2 HC	Relative ACC: range and freq	Thresholds on freq; TP, TN, FP, FN	71% sens., 100% spec. for RT; 89% sens., 97% spec. for PT
Bazgir et al. 2015	Smart phone	custom made glove case	100 Hz	Resting task (1 min); Postural task	Train set: 43 PwPD; Test set: 9 PwPD	Freq (PSD, F50, SF50, F0)	ANN	89.6% sens., 90.6% spec., 91% acc. for UPDRS correlation.
Alhamid, Alamri, and El Saddik 2010	ACC	Hand	100 Hz	Reaching task: handle a cup while moving the hand between two positions	19 volunteers with/without tremor (included PwPD)	PSD	ACC data Periodiagram	Tremor can be detected with the average curve of periodiagram ACC data
Thanawattano et al. 2015	ACC, GYRO	Index fingertip	125 Hz	Resting task; Kinetic task: finger-to-nose movement (each 10 s)	32 PwPD, 20 ET	Temporal fluctuation, fluctuation ratio of resting to kinetic task	Threshold algorithm	100% acc.
Rigas et al. 2016	Microsoft Band	Wrist	62.5 Hz	Resting task; Postural task; ADL	11 PwPD	Energy, energy ratios, principal components, tremor amplitude, tremor freq	C4.5 DT, 10-fold cross-validation; Pearson coefficient	94% acc. for tremor detection; 85% acc. for RT/PT discrimination. r=0.95 for UPDRS correlation to tremor amplitude. r=0.97 for UPDRS correlation to tremor constancy.
Zhou et al. 2016	ACC, GYRO	Wrist and Finger	100 Hz	Resting task; Postural task and distracting questions (each 60 s)	18 PwPD for RT, 13 PwPD for PT	RMS of: linear ACC, angular velocity and displacement; power distribution	N/A	The PD tremor consist of multiple harmonics which are not sinusoidal
Pierleoni et al. 2014	ACC, GYRO	Wrist	128 Hz	Resting task; Postural task; Kinetic task (finger-to-nose movement) and distracting questions if tremor did not occur (each 60 s)	30 PwPD for UPDRS correlation; 12 PwPD for tremor classification	PSD, F0, F50, SF50	PSD and corrective H factor	100% sens.,100% spec. for UPDRS correlation; 100% sens., 100% spec. for tremor classification
Hossen 2012	ACC, EMG	Not reported	800 Hz	Not specified	Train set: 19 PwPD, 21 ET; Test set: 20 PwPD, 20 ET	Power spectral analysis	Feed forward ANN	90% sens., 85% spec., 87.5% acc. for PD/ET classification
Cavallo et al. 2013	ACC, GYRO	Wrist and Fingertips	100 Hz	Resting task; Postural task (each 10 s)	10 PwPD, 5 HC	PSD	PCA; Pearson coefficient	PCA visually well discriminate PwPD/HC; 0.77<r<0.88 between selected features and UPDRS score

Fukumoto 2014	ACC	Arm	Not reported	Not specified	6 PwPD (L-dopa treatment), 6HC; 10 PwPD (biofeedback)	Tremor freq, tremor power	N/A	Decrease of tremor power (p<0.05) and increase of tremor freq (p<0.05) due to biofeedback and L-dopa treatment
Roy et al. 2011	ACC, EMG	Distal portion of each limb	Not reported	4 h continuously recorded during unscripted and unconstrained activities in a 100 m^2 lab that simulated a studio apartment	Train set: 11 PwPD; Test set: 4 HC, 8 PwPD	Low pass energy, High pass energy, Lag and Height of first peak in autocorrelation of ACC corrected signal	DNN	>90% sens., >90% spec. for moderate and severe levels of tremor and dyskinesia
Ruonala et al. 2014	EMG Biomonitor ME6000, ACC	EMG: biceps brachii (BB) muscle of both hands; ACC: forearm	1000 Hz	7–8 repetitions of biceps flexion/extension with elbow staying sitting, repeated with different DBS settings with randomized order	13 PwPD with DBS	Correlation dimension, Recurrence rate, Wavelet maximum	N/A	No substantial change in tremor or rigidity in patients within the measurement. Some patients did not react to DBS adjustment. Tremor and rigidity generally stronger on the right hand side. The most significant increase relative to optimal setup was observed when the stimulator was turned off

Table 3b. Conference papers about gait and TUG analysis.

Ref	Tech.	Sensors Place	Rec. Freq	Experimental Design	Subjects	Feature extracted	Analysis / Classifiers	Classifier Performance or Findings
Oung et al. 2015	ACC, GYRO	Wrists, lower limbs	100 Hz	Walking >10 m, turn around and return; arising from a chair; supination/pronation hand movement; hand flexion/extension; hand movement; finger tapping (FT); leg movement; toe tapping	15 PwPD, 15 HC	Time domain: mean, variance, SD, integrated FOG, mean absolute value, simple square interval, RMS, v-order 2 and 3, waveform length, average amplitude change, difference absolute SD value, max fractal length. Freq domain: FI, entropy, total power, mean power, mean freq, median freq, peak freq, variance, SD, freq ratio, power spectrum ratio, 1st, 2nd and 3rd spectral moments (for each axis)	10-fold cross validation for SVM with RBF kernel and Probabilistic Neural Network (PNN) with 0.2, 0.06, 0.005 spread factor (η)	For time domain features: 82.84% acc., 83.6% sens., 82.4% spec., 83.23% ROC for SVM; 83.89% acc., 84.76% sens., 83.83% spec., 84.36% ROC for PNN (η=0.2); 83.84% acc., 83.76% sens., 83.17% spec., 83.46% ROC for PNN (η=0.06); 83.59% acc., 84.4% sens., 83.23% spec., 83.82% ROC for PNN (η=0.005). For freq domain features: 88.8% acc., 88.7% sens., 88.15% spec., 88.48% ROC for SVM; 88.44% acc., 87.64% sens., 87.75% spec., 87.7% ROC for PNN (η=0.2); 88.61% acc., 88.67% sens., 88.52% spec., 88.6% ROC for PNN (η=0.06); 87.03% acc., 86.38% sens., 86.5% spec., 86.45% ROC for PNN (η=0.005).
Jarchi et al. 2015	ACC	ear-worn Activity Recognition sensor	Not reported	16 repeated trials of 7 m walkway	10 PwPD with DBS	step freq	RMS	RMS=0.0306
Fatmehsari and Bahrami 2010	ACC, DBS system	Thighs, shanks	Not reported	Walking	9 DBS ON and 9 DBS OFF PwPD, 10 HC	approximate entropy, Hurst exponent and Higuchi Fractal Dimension for evaluating irregularity, predictability and complexity of the gait	Leave one out cross validation, kNN	100% acc. using four gyroscope for HC/PwPD DBS OFF discrimination. 89.47% acc. using four gyroscope for HC/PwPD DBS ON discrimination
Arora et al. 2014	Smartphone with ACC	Not specified	Not reported	Walking 20 steps forward, turn around and return back (1 month controlled study)	10 PwPD, 10 HC	Mean, SD, 25th and 75th percentile, IQR, median, mode, data range, skewness, kurtosis, mean squared energy, entropy, cross correlation ACCx-ACCy, mutual information ACCx-ACCy, cross-entropy ACCx-ACCy, extent in randomness in body motion, instantaneous changes in energy, autoregression coefficent at lag 1, zero-crossing rate, dominant freq, radial distance, polar and azimuth angle	RF, Random Classifier, Conditional Random Classifier	For PD/HC classification: 98.5% sens., 97.6% spec., 98.0% acc. for RF; 50.0% sens., 50.2% spec., 50.1% acc. for Random Classifier; 67.7% sens., 32.6% spec., 49.9% acc. for Conditional Random Classifier
Barth et al. 2013	GYRO	Foot	50 Hz	Template data: 10 m walking. Test data: 30 min of gait recording. Test data, daily activity: walking patterns (regular straight, stairs, 8 shaped circles) and daily life activities (sitting, lying, preparing a sandwich, washing dishes, sweeping).	Template data: 25 HC; Test data: 10 HC, 10 PwPD; Test daily activity: 4	Step recognition using DWT	Not reported	Steps correctly recognized: 97.7% HC, 75.5% PwPD, 86.7% daily activity
Reinfelder et al. 2015	ACC, GYRO	Feet	102.4 Hz	TUG (3 m)	16 PwPD	statistical features (e.g. RMS, kurtosis, skewness, mean, mean Euclidean norm, SD, variance, CV, min and max, zero crossing rate, range, integral, normalized jerk score, jerk score and entropy),	NaiveBayes, kNN, SVM with RBF kernel, RF	56.87% NaïveBayes, 75.41% kNN, 81.8% SVM, 75.03% RF. The total time of the TUG test increased with the severity of the disease according to the UPDRS and HY stages.

						signal energy feature (e.g. PSD, energy ratio and energy in freq band), gait features (e.g. stride time, angle between two consecutive strides)		
Al-Jawad et al. 2012	ACC, GYRO	Lower back	100 Hz	TUG (3 m)	20 PwPD (10 early and 10 late), 10 HC	angular velocity, angle, LDA of the stacked cross correlation between angular rate in pitch axis and the AP ACC with the cross correlation between the vertical (VT) and ML ACC (DTW-based method)	LDA; Wilcoxon rank sum test; DTW-based algorithm	Differences in: Si2St between HC/earlyPD (p=0.03); first TUG turn between HC/latePD (p=0.0001), HC/earlyPD (p=0.02), earlyPD/late PD (0.007); second TUG turn between HC/latePD (p=0.0001), earlyPD/late PD (p=0.018); overall course between HC/latePD (p=0.0033), earlyPD/late PD (p=0.023). 3D DTW performed better than 1D
Caldara et al. 2014	ACC, GYRO, Visual feedback	Each limb and chest	50 Hz	Extended TUG test (10 m)	13 PwPD, 4 HC	posture, gait direction, turning, exercise duration, intermediate time by spine, oscillation, tremors, asymmetries by forearms, gait quality, FOG, bradykinesia from legs	N/A	The system is able to monitor a considerable amount of parameters as asymmetries during gait, posture, tremors and total and intermediate times of the exercise execution.
Barth et al. 2011	ACC, GYRO	Foot	100 Hz	10 m walking; circling the foot (20 s); heel toe tapping (20 s)	14 early and 13 mid PwPD, 16 HC	Step duration, rise/fall gradient of swing phase, SD of min, max-min difference, variance, integral, dominant freq, energy ratio, energy in band 0.5-3 Hz and in band 3-8 Hz	Boosting with Decision Stump, LDA, SVM	88% sens., 86% spec. for early diagnosis (early PwPD/HC). 100% sens., 100% spec. for therapy monitoring (mid PwPD/HC, early PwPD/mid PwPD)
Salarian et al. 2009	GYRO	Shanks, sternum	200 Hz	3 turning trials. Walking on a straight, 7 m long, clearly marked pathway. Subjects walked at their normal speed, turn around right after passing the tape at the end of the pathway and return back	14 PwPD (de-novo), 12 HC	Peak angular velocity, duration, steps, average step time, maximum step time, step before turn, number of double steps	Wilcoxon test; ICC; Coefficient of determination (R^2)	R^2=0.9989. Significant differences between PwPD/HC in duration of turns, number of double-steps and duration of last step before turn. PwPD were slower and had more double-steps. ICC>0.85 for duration of turns, peak angular velocity of trunk and the duration of the last step before turn
Tien, Glaser, and Aminoff 2010	ACC, GYRO	Foot	Not reported	Walking task along a predetermined path along a hallway	21 significant gait disturbance PwPD, 24 no significant gait disturbance PwPD, 24 HC	67 features including: ROM, max angles of dorsiflexion and plantar flexion, SD of plantar flexion, roll, pitch and yaw angles, cadence	PCA; SVM (RBF kernel, 10-fold cross validation)	Prediction performance metrics for cases of equal or varying misclassification costs: 93.9% sens., 95.8% spec., 4.2% false positive rate, 97.7% prec.. Prediction performance for multi-class classification task (class recall/class prec.): PwPD with gait disturbance 52.4/84.6%, PwPD without gait disturbance 66.7/64.0%, HC 91.7/71%
Cabestany et al. 2013	ACC, GYRO	Waist	80 Hz	Short controlled tests (e.g. walking through a door, making turns) and free activity monitoring	90 PwPD	gait speed, step/stride length, FOG, dyskinesia	PCA; SVM	Numerical results not reported
Cancela et al. 2011	ACC, GYRO	Each limb and belt	62.5 Hz	Move freely and perform daily activities in ON/OFF state	10 PwPD	gait speed, step/stride length, step freq, entropy, arm swing	N/A	Step freq, stride length, entropy and arm swing presented a significant variation between ON/OFF in all the patients. No direct correlation between variation in the magnitudes and in UPDRS

Table 4b. Conference papers about FOG analysis.

Ref	Tech.	Sensors Place	Rec. Freq	Experimental Design	Subjects	Feature extracted	Analysis / Classifiers	Classifier Performance or Findings
Cole, Roy, and Nawab 2011	ACC, EMG	Forearm, shank	1000 Hz	Unscripted and unconstrained ADL. Train: 6 min recording including 20 FOG episodes. Test: 2 h recording including 87 FOG episodes	Train set: 6 PwPD; Test set: 4 PwPD, 2 HC	Data from ACC and EMG, FOG episodes duration	DNN	82.9% sens., 97.3% spec. for FOG events detection
Bächlin et al. 2009	ACC, earphones	Thigh, shank, trunk	64 Hz	i) walking back / forth in a straight line, including several 180° turns; ii) random walking in a reception hall space, including a series of initiated stops and > 6 several 360° turns. iii) walking simulating ADL (entering / leaving rooms, walking to the lab kitchen, getting something to drink, returning to the starting room with the cup of water). (5-10 min for task, twice: with/without external cues)	10 PwPD with FOG (8 with FOG during the study) (8 PwPD were in OFF state, 2 PwPD were in ON state)	FI, PSD	N/A (Comparison to video analysis)	73.1% sens., 81.6% spec. for the online FOG detection
Handojoseno et al. 2012	4 channel wireless EEG system	Head	500 Hz	Structured series of video-recorded TUG tasks The features were measured during normal walking, FOG onset, FOG	10 PwPD with significant FOG	Wavelet energy (WE), relative WE (α, β, γ, δ, θ), total wavelet entropy (WEE)	Wilcoxon Sum Rank Test. Back Propagation NN	72.0% sens., 77.2% spec., 75% acc. for normal/onset classification. 71.2% sens., 77.2% spec., 73.9% acc. for normal/FOG classification. p<0.05 in almost all features between normal/onset, normal/FOG, onset/FOG
Handojoseno et al. 2013						total wavelet cross spectrum (WCS) of EEG α, β, γ, δ, θ, centroid freq WCS. Mean, SD, kurtosis, max, min, skewness	Wilcoxon Sum Rank Test. MLPNN, kNN (5 to 40 nearest neighbors)	Altered pattern of synchronisation in the θ sub-band during transition from walking to FOG and during FOG. Up to 87% sens., 73% acc. for FOG detection
Handojoseno et al. 2014						Directed transfer function (DTF), direct DTF (dDTF), partial directed coherence (PDC), squared generalized PDC (sGPDC)	Wilcoxon Sum Rank Test; MLPNN	Abnormal EEG hyperconnectivity in the frontal region during FOG episodes. FOG detection: mean 69.5% sens., 70.5% spec., 70.0% acc. using DTF, dDTF, PDC, sGPDC; 82.2% sens., 77.3% spec., 78.0% acc. using sGPDC
Mazilu et al. 2014	ACC, GYRO, GaitAssist system / smartphone	Ankles	32 Hz	24h recording data	18 PwPD	Freq features: total power, locomotion band power, freezing band power, FI	C4.5 DT	94.94% hit rate and 94% spec.
Mazilu and Hardegger 2012	ACC, GYRO, Smart phone	Thigh, shank, lower back	64 Hz	i) walking back / forth in a straight line, including several 180° turns; ii) random walking in a reception hall space, including a series of initiated stops and > 6 several 360° turns. iii) walking simulating ADL (entering / leaving rooms, walking to the lab kitchen, getting something to drink, returning to the starting room with the cup of water). (10-15 min, twice)	10 PwPD regularly experienced FOG	Mean, SD, variance, entropy, energy, FI, power	Naïve Bayes, MLPNN, AdaBoost C4.5, Bagging C4.5, C4.5 and RF with 10-fold cross validation	99.69% sens., 99.96% spec. for FOG events detection from C4.5. Mean latency of 0.34 s.

Table 5b. Conference papers about postural instability.

Ref	Tech.	Sensors Place.	Rec. Freq	Experimental Design	Subjects	Feature extracted	Analysis / Classifiers	Classifier Performance or Findings
Masu et al. 2016	ACC	C7, L4 vertebras	Not reported	Standing (30 s)	19 mild and 24 severe PwPD, 17 young and 17 elderly HC	150 features about postural angles included range, average, variance and skewness	Steel- Dwass and Kruskal-Wallis tests; SVM with cross-validation	Acc.: 81.0% Severe PD/Mild PD; 90.0% Severe PD/Elderly HC; 77.8% Mild PD/Elderly HC; 92.9% Severe PD/Young HC; 89.5% Mild PD/Young HC; 80.6% Elderly HC/Young HC
Pasluosta et al. 2015	ACC, GYRO	Feet	102.4 Hz	i) 4x10 m-walk at a self-selected comfortable speed; ii) 2x10 m-walk Stop and Go (SG) at a self-selected comfortable speed, 3 times stop and resume the walking; iii) Heel-to-Toe tapping alternately on the floor (20 s each foot) while sitting in a chair; iv) Circling (CL) foot movements above the floor while sitting (20 s each foot)	139 PwPD: 47 bradykinetic, 31 tremor-dominant (TD), 61 both symptoms	Mean and variability of: stride time, swing time, stance time, stride length. Number of strides, angle heel-strike, angle toe-off, max toe clearance, cadence, estimated distance, gait velocity, entropy, mean value, max and min values, RMS, kurtosis, skewness, dominant freq, power in range [0.5-3]Hz, power in range [3-8]Hz, energy ratio, signal energy	SVM (RBF kernel, 5 fold cross validation)	0.75 acc. for complete dataset. 0.79 acc. for PwPD with both symptoms using only CL data. 0.75 acc. for bradykinetic PwPD using only CL data. 0.70 acc. for tremor PwPD using only SG data

Table 6b. Conference papers about upper limbs motion analysis.

Ref.	Tech.	Sensors Place.	Rec. Freq	Experimental Design	Subjects	Feature extracted	Analysis / Classifiers	Classifier Performance or Findings
Eskofier et al. 2016	ACC	Forearm	50 Hz	Finger-to-nose, pronosupination (twice, 15 s)	10 PwPD	Energy, max, min, mean, variance, skewness, kurtosis, spectral analysis	AdaBoost.M1, PART, kNN, SVM (leave-one-out cross validation), deep learning	Acc. 86.3% AdaBoost.M1, 81.7% PART, 67.1% kNN, 85.6% SVM, 90.9% Deep Learning for bradykinesia assessment
Jia et al. 2014	ACC, wrist-watches (pressure sensor, ACC)	Wrists	20 Hz	FT, hands opening/closing, pronosupination	12 PwPD, 12 HC	Range, SD, entropy, time and max freq	N/A (Histograms)	83.3% sens., 75% spec. for SD for PD/HC classification
Hoffman and McNames 2011	ACC, GYRO	Index finger	128 Hz	FT as pad-pad and tip-knuckle, pronosupination (each 15 s)	11 PwPD, 35 HC	Angular velocities	Adaptive filtering algorithms: Ordinary Least Squares (LS), Least Mean Square (LMS), Recursive LS (RLS), Kalman Filter (KF)	AUC 0.781, p=0.026 for KF in pad-pad FT; AUC 0.828, p=0.009 for LMS in tip-knuckle FT; AUC 0.869, p=0.036 for RLS in pronosupination
Fukawa et al. 2007	ACC, 2 touch sensors	Index finger and thumb	0.1 ms	FT (60 s)	17 PwPD, 44 HC	SD of FT intervals, average of the max velocity of the single FT, average of the max amplitude during the single FT, average of contact force of a single FT	ANN	UPDRS FT score could be estimated with the proposed ANN. Results reported only in box-plots
Okuno et al. 2007					16 PwPD, 27 HC	Average FT contact force	N/A	The contact force decreased with increasing the score of UPDRS FT test. Results reported only in box-plot
Djurić-Jovičić et al. 2014	GYRO	Fingertip of index finger	Not reported	FT (15 s)	10 PwPD, 10 HC	Cross-sectional areas (CWT analysis)	t-Test, Mann-Wilcoxon test, quadratic and nearest mean scaled classifiers	94.4% acc. for quadratic classifier for PD/HC classification
Barth et al. 2012	ACC, Biometric Smart Pen	Hand	1000 Hz	On paper: drawing 12 circles at the same place; tracing 4 preprinted spirals; tracing 4 preprinted meanders. In the air: drawing 12 circles around a virtual point; performing pronosupination movements (20 s); performing FT on the pen (20 s)	18 PwPD, 17 HC	Mean, variance, regression line gradient, SD, range, autocorrelation max, integral, RMS, dominant freq, energy ratio, energy in freq band, regression line of windowed energy in freq band, fall gradient of stance phase	AdaBoost, 30 iterations, CFS linear forward feature selection	89% classification rate, 94% sens., 83% spec. for PwPD/HC classification

Table 7b. Conference papers about rigidity, arms swing and leg agility analysis.

Ref.	Symptom	Tech	Sensors Place.	Rec. Freq	Experimental Design	Subjects	Feature extracted	Analysis / Classifiers	Classifier Performance or Findings
Giuberti et al. 2014	LA	ACC, GYRO	Thighs	102.4 Hz	10 repetitions of LA per leg	1st study: 1 PwPD and 1 HC. 2nd study: 24 PwPD	angular amplitude of thigh inclination, angular speed of execution, pause of execution, regularity between consecutive repetitions, relative difference of the angular amplitude and of the angular speed between left and right legs, repetition freq, power spectrum	PCA; NCC, kNN and SVM both on original data and PCA data	Correlation between heels' optical data and thighs' inertial data (r>0.98). Relative difference between RLA and LLA around 4% for HC and 6% for PwPD. Best classifier: kNN, k=3 which maximize the AUC of the CDFs and minimize the error between actual UPDRS score and estimated UPDRS score

Table 8b. Conference papers about motor fluctuations analysis.

Ref.	Tech.	Sensor Place.	Rec. Freq	Experimental Design	Subjects	Feature extracted	Analysis / Classifiers	Classifier Performance or Findings
Samà et al. 2012	ACC, GYRO	Lower back	200 Hz	Training protocol: walking three times in a straight line of ~5 m in lab. Testing protocol: walking in a straight line, walking over an inclined plane, carrying a heavy object, setting a table and going upstairs and downstairs, walking outside for at least 15 min.	Train set: 10 PwPD; Test set: 10 PwPD	Spectral analysis	Thresholds; SVM (10-fold cross validation, RBF kernel); AUC	89% sens., 78% spec. for dyskinesia detection; 90% sens., 84% spec. and 94% acc. for gait detection; 0.83<AUC<0.85 for ON/OFF states detection; 0.91<AUC<0.94 for ON/OFF states detection averaging 5 strides
Ruonala et al. 2015	Biomonitor ME6000, ACC	Chest	1000 Hz	Three 5 min ECG measurements: before Ldopa administration (MEDoff), 30 min after the administration (MED30), 60 min after the administration (MED60)	11 PwPD	Time-domain: Mean RR, SDNN, RMSSD, pNN50, HRV triangular index and triangular interpolation of N-to-N interval histogram. Freq-domain: Welchs periodogram, LF and HF band powers, LF/HF power ratio, total spectral power. Others: Poincaré plot indexes for short-term variability (SD1) and overall variability (SD2)	Median, IQR, Wilcoxon signed rank test	significant decrease in RMSSD, SD1 and HF power between MEDoff/MED30
Hssayeni, Burack, and Ghoraani 2016	ACC, GYRO	Trunk, wrist, ankle (side more affected)	Not reported	drinking from a cup, walking, unpacking groceries, sitting still with arms resting in the lap, cutting food, dressing, and hair brushing (30-60 s each)	12 PwPD	signal power, jerk, entropy, peak-to-peak, correlation coefficient	K-means	70.57% sens., 86.93% spec. and 75.96% acc. for ON/OFF states detection
Pastorino et al. 2011	ACC, GYRO	Each limb and belt	62.5 Hz	Training: walking, lying on bed, sitting on a chair, drinking a glass of water, opening / closing a door. Testing: unsupervised environment during a week. 8h per day	Train set: 20 PwPD; Test set: 6 PwPD	Range and RMS	SVM; ICC	74.4±14.9% acc. to UPDRS correlation for bradykinesia. 0.90 ICC
Rahimi et al. 2011	ACC, GYRO	Each limb, trunk, pelvis, head	100 Hz	1st protocol: walking and turning, sitting and rising from a chair, figure 8 turns, and reaching tasks. 2nd protocol: free daily activity (1 h)	11 PwPD	Inter-trial variability, inter-subject variability, inter-task variability	PCA	Very large variability among PwPD
Tsipouras et al. 2011	ACC, GYRO	Wrists, legs, waist, chest	62.5 Hz	To act freely, speak and make voluntary movements (subject seated)	24 PwPD (10 LID), 5 HC	Mean, SD, mean entropy, signal energy in different bands, spectral entropy, spectral SD from each axis of each sensor	ANN leave-one-patient-out cross validation	83.3% acc. from wrists; 85.3% acc. from legs; 84.3% acc. from chest; 84.2% acc. from waist. For LID severity assessment

Table 9b. Conference papers about home and long-term monitoring.

Ref.	Tech.	Sensors Place.	Rec. Freq	Experimental Design	Subjects	Feature extracted	Analysis / Classifiers	Classifier Performance or Findings
Cancela et al. 2010	ACC	Each limb, trunk and waist	Not reported	Daily basic activities: walking, lying, sitting, drinking a glass of water, opening/closing a door	20 PwPD	RMS, range, sample entropy, approximate entropy, cross-correlation	kNN, ParzencParzen, ParzendcParzen density based, DT, Bpxnc Train NN, SVM	70%-86% acc. for bradykinesia severity
Khan et al. 2014	ACC, BioMotion Suite	Waist	32 Hz	1 hour monitoring performing daily activities	12 PwPD (mid /late)	The moving average of the SD in the accelerometer; an assessment of the first peak in the signal's power spectrum; wavelet decomposition of the signal	Multi-class SVM with RBF kernel	72% overall acc. to detect normal, tremor (hand or leg) and dyskinesia (majority of the errors due to falsely detecting tremor)
Lambrecht et al. 2014	ACC, GYRO	Hand, distal forearm, proximal forearm, arm	100 Hz	ADLs: answering a phone, buttoning a coat, brushing teeth, combing hair, cutting a steak, dialing a phone number, eating, opening/closing a door or a container, reading a book, signing a form, drinking. Functional tasks: wrist flexion, elbow flexion, wrist circumduction, pronation/supination, resting task	13 PwPD and ET	Sum of: max values (x,y,z), RMS (x,y,z), RMS square, variance (x,y,z), eigenvalues of covariance matrix, PC coefficients	Ranking	50% acc. for resting task; 91.77% acc. for all the other tasks with 4 IMU. 98.3% acc. for all the other tasks with 3 IMU (proximal forearm excluded)
